# Inhibition of α-Glucosidase, Acetylcholinesterase, and Nitric Oxide Production by Phytochemicals Isolated from *Millettia speciosa*—In Vitro and Molecular Docking Studies

**DOI:** 10.3390/plants11030388

**Published:** 2022-01-30

**Authors:** Nguyen Ngoc Tuan, Huong Nguyen Thi, Chau Le Thi My, Tang Xuan Hai, Hieu Tran Trung, Anh Nguyen Thi Kim, Thanh Nguyen Tan, Tan Le Van, Cuong Quoc Nguyen, Quang De Tran, Ping-Chung Kuo, Quang Le Dang, Tran Dinh Thang

**Affiliations:** 1 Institute of Biotechnology and Food Technology, Industrial University of Ho Chi Minh City, Ho Chi Minh 71408, Vietnam; nguyenngoctuan@iuh.edu.vn (N.N.T.); nguyenthikimanh@iuh.edu.vn (A.N.T.K.); 2 School of Natural Sciences Education, Vinh University, Vinh City 43100, Nghean, Vietnam; nguyenthihuongtn@hdu.edu.vn (H.N.T.); trantrunghieu94tc@gmail.com (H.T.T.); 3 Faculty of Natural Sciences, Hong Duc University, Thanh Hoa 40100, Vietnam; 4 School of Chemistry, Biology and Environment, Vinh University, Vinh City 43100, Nghean, Vietnam; lemychau83@gmail.com (C.L.T.M.); nguyentanthanhvn@gmail.com (T.N.T.); 5 Nghe An Obstetric & Paediatric Hospital, Vinh City 43100, Nghean, Vietnam; bstangxuanhai@gmail.com; 6 Faculty of Chemical Engineering, Industrial University of Ho Chi Minh City, Ho Chi Minh 71408, Vietnam; levantan@iuh.edu.vn; 7 Department of Chemistry, College of Natural Sciences, Can Tho University, Can Tho 90000, Vietnam; ncquoc99@gmail.com (C.Q.N.); tqde@ctu.edu.vn (Q.D.T.); 8 School of Pharmacy, College of Medicine, National Cheng Kung University, Tainan 70101, Taiwan; z10502016@ncku.edu.tw; 9 R&D Center of Bioactive Compounds, Vietnam Institute of Industrial Chemistry, Hanoi 10000, Vietnam; 10 Institute for Tropical Technology, Vietnam Academy of Science and Technology, Hanoi 10000, Vietnam

**Keywords:** *Millettia speciosa*, molecular docking, NO production, anti-glucosidase, anti-acetylcholinesterase

## Abstract

The phytochemical constituents from the roots of *Millettia speciosa* were investigated by chromatographic isolation, and their chemical structures were characterized using the MS and NMR spectroscopic methods. A total of 10 compounds, including six triterpenoids, two flavonoids, and two phenolic compounds, were identified from the roots of *M. speciosa*. Out of the isolated compounds, eight showed inhibitory effects on NO production in lipopolysaccharide (LPS)-stimulated RAW 264.7 cells, with IC_50_ values ranging from 43.9 to 449.5 µg/mL. Ursane-type triterpenes significantly suppressed NO production compared to the remaining compounds. In addition, these compounds also exhibited remarkable inhibitory effects on α-glucosidase. Among the tested compounds, **4**, **5**, and **10** exhibited excellent α-glucosidase inhibition, with IC_50_ values ranging from 1.1 to 2.2 µg/mL. Almost all of the test compounds showed little or no acetylcholinesterase inhibition, except for **5,** which showed moderate anti-acetylcholinesterase activity in vitro. The molecular docking study of α-glucosidase inhibition by **3**–**5** and **10** was conducted to observe the interactions of these molecules with the enzyme. Compounds **4**, **5**, and **10** exhibited a better binding affinity toward the targeted receptor and the H-bond interactions located at the entrance of the enzyme active site pocket in comparison to those of **3** and the positive control acarbose. Our findings evidence the pharmacological potential of this species and suggest that the phytochemicals derived from the roots of *M. speciosa* may be promising lead molecules for further studies on the development of anti-inflammatory and anti-diabetes drugs.

## 1. Introduction

*Millettia* (Leguminosae) is a genus of about 200 species found in tropical and subtropical regions of Africa and Asia [1]. The chemical composition of *Millettia* is characterized by flavonoids, including chalcones, isoflavones, and triterpenoids [2,3]. Most of these plants are known for their folk medicinal applications. For example, *M. speciosa* and *M. nitida* var. *hirsutissima* are used in traditional medicine to alleviate dysmenorrhea, rheumatic, and paralysis [4,5]. *Millettia griffoniana* is employed orally for the treatment of boils, inflammation, amenorrhea, menopausal syndromes, sterility, and insect bites [6,7]. *Millettia oblata* has therapeutic uses for stomachache, cough, and swollen body, while *M. usaramensis* used as a remedy against snake bites [2].

*Millettia speciosa* Champ. (syn. *Callerya speciosa*) is a sub-shrub plant that is extensively used as a tonic and found primarily in the Chinese regions of Guangdong and Guangxi. Its roots are used to alleviate lumbago, as well as to strengthen bones and muscles [8,9,10]. It was commonly used as an edible plant in local regions, such as stewed chicken or soup, with the benefit of strengthening bones and muscles. *Millettia speciosa* widely grows in various provinces of Vietnam, including Tuyen Quang, Bac Can, Quang Ninh, Phu Tho, Bac Giang, Ha Noi, Lang Son, and Ha Nam [11]. *Millettia speciosa* roots have traditionally been used in Vietnamese traditional medicine to treat rheumatism, chronic bronchitis, and hepatitis [12]. Polysaccharides of *M. speciosa* have been considered to be the main active constituents and immunomodulatory agents for functional foods and traditional medicine [13]. A polysaccharide fraction (MSCP2) extracted from the roots of *M. speciosa* was reported to stimulate cytokine and NO production and suggested to be a promising immunomodulatory ingredient [14]. Six fractions of heteropolysaccharides derived from the roots of *M. speciosa* were effective at enhancing intestinal health, increasing immune-related cytokine production, ameliorating body weight, and protecting immunological organs [13].

Previous phytochemical studies have reported that the root extract of *M. speciosa* contains various constituents, including alkaloids, oleanane-type triterpene saponin, flavonoids, isoflavones, rotenoids, lignans, chalcones, and phenolic glycosides [5,15,16,17,18,19,20,21,22]. Using UPLC-Q-TOF-MS/MS, Dandan and Xianrui characterized 21 isoflavones and 4 isoflavanones in the roots of *M. speciosa* [23].

Docosanoic acid, tetracosane, octadecane, hexacosanoic acid, *β*-sitosterol acetate, *β*-sitosterol, syringin, maackiain, formononetin, ψ-baptigenin, rotundic acid, pedunculoside, and daucosterol were among the 13 phytochemicals discovered by Ding et al. [9]. An oleanane-type triterpenoid, 22*β*-acetoxy-3*β*,24-dihydroxy-12-oleanen-30-oic acid, together with medicarpin, maackiain, and *β*-sitosterol were identified from the ethyl acetate extract of the roots of *M. speciosa* growing in Vietnam [12]. However, the pharmaceutical properties of many constituents of this species were not fully described. There are also a few reports of the biological data for the secondary metabolites of Vietnamese *M. speciosa*. In our ongoing study of bioactive compounds from medicinal plants growing in Vietnam, we focused on the isolation of secondary metabolites from the roots of *M. speciosa* and characterized their chemical structures by the NMR and MS spectroscopic methods. The inhibition of anti-glucosidase, anti-acetylcholinesterase, and NO production by all isolated compounds was evaluated in vitro. In addition, a molecular docking study of α-glucosidase inhibition by potent inhibitors was conducted to understand the interaction between the inhibitors and the enzyme.

## 2. Results

### 2.1. Structural Characterization of the Isolated Compounds

**Figure 1:**
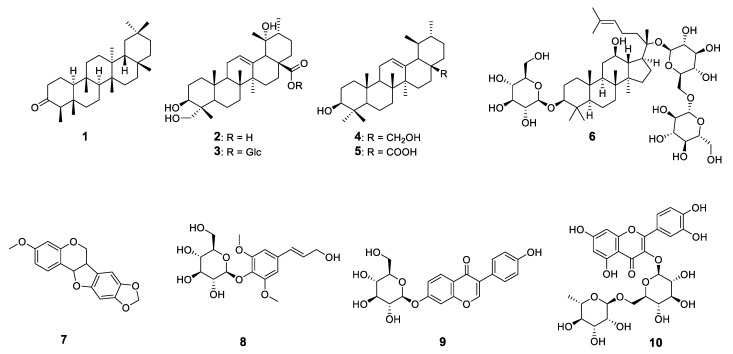
Chemical structures of the isolated compounds from the roots of *Milletia speciosa*. Compounds—**1**: friedelin; **2**: rotundic acid; **3**: pedunculoside; **4**: uvaol; **5**: ursolic acid; **6**: gypenoside XVII; **7**: pterocarpin; **8**: syringin; **9**: daidzin; **10**: rutin.

A total of 10 compounds, including six triterpenoids, two flavonoids, and two phenolic compounds were identified from the roots of *M. speciosa* (Figure 1).

Compound **6** was obtained as a white powder. The structure of Compound **6** was confirmed and verified by its HR-ESI-MS and 1D- and 2D-NMR (Data S1—Supplementary Materials). The ^1^H-NMR spectrum, the protons of 3-*O*-glucopyranosyl, and 20-*O*-glucopyranosyl moiety appeared at 4.33 (1H, *t*, *J* = 7.0 Hz, C_1′_-H), 4.59 (1H, *d*, *J* = 8.0 Hz, C_1″_-H), and 4.32 (1H, *t*, *J* = 7.0 Hz, C_1‴_-H), respectively. The anomeric proton signals showed that Compound **6** harbored three *β*-D-glucoses moieties. In addition, a comparison of the ^13^C-NMR spectrum of Compound **6** with that of gypenoside XVII suggests that Compound **6** is identical to 3-*O*-[*β*-D-glucopyranosyl]-20-*O*-[*β*-D-glucopyranosyl-(6→1)-*β*-D-gluco-pyranosyl]-20(*S*)-protopanaxadiol [24].

Compound **8** was isolated as a white amorphous powder, and its mass spectral data suggest the molecular formula is C_17_H_24_O_9_. The ^1^H-NMR spectrum of **1** indicates the presence of a phenylpropanoid skeleton at *δ*_H_ 6.48 (1H, *d*, *J* = 16.0 Hz, C_7_-H), 6.35 (1H, *dt*, *J* = 11.5; 5.0 Hz, C_8_-H), and 4.30 (2H, *dd*, *J* =11.5; 5.5 Hz, C_9_-H), a glucose moiety at *δ*_H_ 3.01–3.73 (6H, *m*, C_2′/3′/4′/5′/6′_-H), and an anomeric proton at *δ* 4.91 (1H, *d*, *J* = 7.5 Hz, C_1′_-H). The coupling constant of *J* = 16.0 Hz is attributable to one pair of *trans* protons, which is the hallmark of cinnamic acid derivatives. *m*-substituted aromatic ring system signals were observed at *δ*_H_ 6.72 (2H, *s*, C_3/5_-H), and two methoxy groups were revealed at 3.76 (6H, *s*, 2- and 6-OCH_3_). In the ^13^C NMR data, a glucose moiety between *δ*_C_ 60.9 (C_6″_) and 76.6 (C_5″_), and an anomeric carbon signal at *δ*_C_ 104.5 (C_1″_) were confirmed. The signal at *δ*_C_ 56.4 (C_2/6_-OCH_3_) indicates two methoxy carbons. Finally, the glucosyl C_1′_-hydrogen atom (*δ*_H_ 4.91) correlated with C_1_ (*δ*_C_ 133.9) of the phenylpropanoid unit in the HMBC spectrum. On the basis of the 1D- and 2D-NMR experiments, Compound **8** was assumed to be syringin [25].

Compound **9** was obtained as a yellow-brown powder. The ESI-MS of Compound **9** showed *m*/*z* 417.2 [M + H]^+^ and was established to be C_21_H_21_O_9_. The signals of the aromatic protons were registered at *δ*_H_ 7.41 (2H, *d*, *J* = 8.0 Hz, C_2′/6′_-H) and 6.82 (2H, *d*, *J* = 8.0 Hz, C_3′/5′_-H). They also exhibited signals at *δ*_H_ 8.05 (1H, *d*, *J* = 9.0 Hz, C_8_-H), and 7.23 (1H, *J* = 2.0 Hz, C_6_-H). In addition, a proton signal at C-5 at *δ*_H_ 7.15 (1H, *J* = 1.5 Hz, C_5_-H) was observed. In line with other studies on the ^13^C-NMR spectra of flavonoids, the carbon chemical shifts of the aglycone skeleton of Compound **9** were consistent with their assignments. The ^13^C NMR chemical shift data of the glucose moiety of daidzin were clearly indicated by analysis of the ^13^C NMR spectra, especially by the resonances for C-1″, C-2″, C-3″, C-4″, C-5″, and C-6″ (*δ*_C_ 100.0, 73.1, 76.5, 69.6, 77.2, and 60.6, respectively). The location of the *β*-glucosyl unit was determined by ^3^*J*-HMBC correlations between H-1′ and C-7. Thus, the structure of **9** was established as daidzin (daidzein-7-O-*β*-D-glucopyranoside) [26].

Compound **10** was obtained as a yellow powder. The ^1^H-NMR spectrum of Compound **10** showed the characteristic signals of the quercetin skeleton—*δ*_H_ 6.26 (*d*, *J* = 2.1 Hz, C_6_-H), 6.45 (*d*, *J* = 2.1 Hz, C_8_-H), 7.68 (*dd*, *J* = 8.4, 2.2 Hz, C_6_-H), 6.92 (*d*, *J* = 8.4 Hz, H-5), and 7.71 (*d*, *J* = 2.2 Hz, H-2). In the HMBC spectrum, the rhamnosyl C1-hydrogen atom (*δ*_H_ 4.56) correlated with the glucosyl C6′ atom at *δ* C 68.5, indicating a rutinosyl moiety. Finally, the glucosyl C1′-hydrogen atom (*δ* _H_ 5.15) correlated with C3 (*δ*_C_ 135.62) of the flavonoid unit in the HMBC spectrum. The analysis of the one- and two-dimensional ^1^H- and ^13^C-NMR spectra of **10** and the comparison with the values found in the literature [27] led to the assignment of Compound **10** as quercetin-3-*O*-rutinoside (rutin).

### 2.2. In Vitro Biological Activities of the Isolated Compounds from Millettia speciosa

In this study, the in vitro anti-glucosidase, anti-AChE, and NO production inhibitory activities of the phytochemical constituents derived from the roots of *M. speciosa* growing in Vietnam were investigated. 

**Figure 2:**
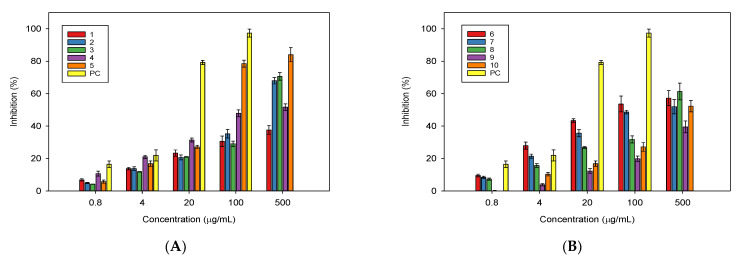
NO production inhibition of the isolated compounds from *Millettia speciosa*. Compounds—(**A**): (**1**: friedelin; **2**: rotundic acid; **3**: pedunculoside; **4**: uvaol; **5**: ursolic acid); (**B**): (**6**: gypenoside XVII; **7**: pterocarpin; **8**: syringin; **9**: daidzin; **10**: rutin). The test compounds were evaluated for their inhibition at a concentration range of 0.8–500 µg/mL. PC: positive control treated with L-NMMA at a concentration range of 0.8–100 µg/mL.

A Griess assay was used to assess the inhibition of NO production in lipopolysaccharide (LPS)-stimulated RAW 264.7 cells by the isolated compounds at concentrations ranging from 0.8 to 500 µg/mL. The results of the NO production assay for the tested compounds indicate that Compounds **2**–**6** displayed moderate activity, followed by Compounds **7**, **8**, and **10** (Figure 2).

Compounds **1** and **9** possessed weak activity for NO production; at 500 µg/mL, their inhibitions for NO production were only 37.6% (Figure 2A) and 39.5% (Figure 2B), respectively. Ursolic acid (**5**) was found to be the best inhibitor for NO production. At 500 µg/mL, this substance caused inhibition of 84.0% (Figure 2A); it also had the smallest IC_50_ of 43.9 µg/mL in comparison to the other constituents (Table 1). Gypenoside XVII (**6**) possessed an IC_50_ of 93.9 µg/mL, followed by Compounds **7**, **2**, **4**, and **3,** with IC_50_ values of 228.9, 241.33, 246.49, and 273.10 µg/mL, respectively (Table 1). As expected in the experiment treated with L-NMMA, the positive control showed good inhibition for NO production, with an IC_50_ of 8.6 µg/mL (Table 1).

**Figure 3:**
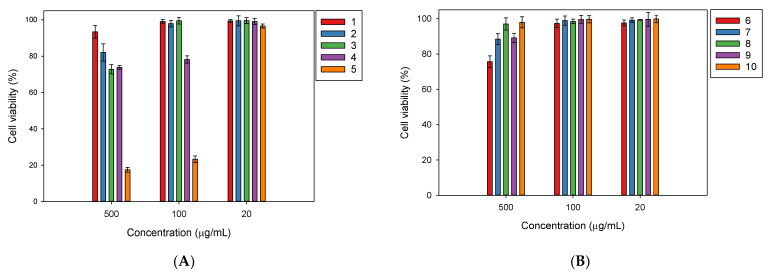
Viability of RAW264.7 cell line treated with the isolated compounds from *Millettia speciosa*. (**A**): (Compounds—**1**: friedelin; **2**: rotundic acid; **3**: pedunculoside; **4**: uvaol; **5**: ursolic acid); (**B**): (**6**: gypenoside XVII; **7**: pterocarpin; **8**: syringin; **9**: daidzin; **10**: rutin). The cells were incubated at 37 °C under CO_2_ atmosphere.

In comparison, all of the test compounds did not show cytotoxicity against RAW 264.7 cells in the cell viability assay, except for Compound **5**, which reduced the cell growth by 23.3% at 100 µg/mL (Figure 3).

The isolated compounds from *M. speciose* were tested for their inhibitory α-glucosidase activity at a concentration range of 1–256 µg/mL. In particular, Compounds **4**, **5**, and **10** show the best α-glucosidase inhibitions, with IC_50_ values of 2.0, 1.1, and 2.2 µg/mL (Table 2). At 256 µg/mL, **4**, **5**, and **10** inhibited α-glucosidase, with inhibitions percentages of 92.5, 99.0, and 85.0%, respectively, even though **5** induced 91.5% inhibition for α-glucosidase at 4 µg/mL (Table S1—Supplementary Materials). Compared to the positive control acarbose (IC_50_ = 169.8 µg/mL), their activity was extremely significant. Compound **3** (pedunculoside) also caused inhibition of 60.5% for α-glucosidase at 256.0 µg/mL and an IC_50_ of 184.9 µg/mL (Table 2 and Table S1—Supplementary Materials). Nevertheless, the remaining compounds, **1**, **2**, and **6**–**9,** showed little to no inhibition for α-glucosidase at the test concentration range. At 256.0 µg/mL, the α-glucosidase inhibitions of those compounds were determined to be 23.0% for **1**, 33.0% for **2**, 34.0% for **6**, 25.0% for **7**, 12.0% for **8**, and 22.0% for **9**, respectively (Table S1—Supplementary Materials).

In the bioassay of AChE inhibition, rutin (**10**) was found to inhibit AChE, with an IC_50_ of 256.0 µg/mL, and Compound **5** was found to be the strongest inhibitor of AChE, with an IC_50_ of 8 µg/mL; however, these inhibitions were much weaker compared to the positive control of donepezil (IC_50_ = 0.025 µg/mL). The other compounds did not display any significant AChE inhibitions in the test concentration range (Table S2—Supplementary Materials).

### 2.3. Docking Study for α-Glucosidase Inhibition by Compounds ***3**–**5*** and ***10***

**Figure 4:**
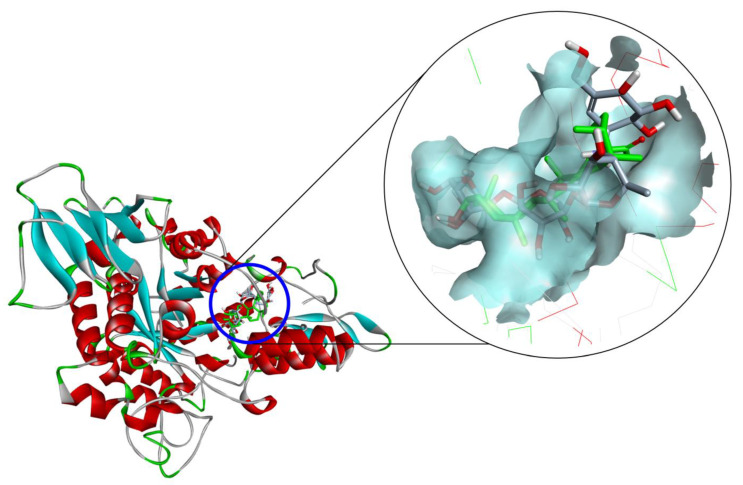
Compound **5** (green—most active) and acarbose (gray—control) at the active site of α-glucosidase.

From the α-glucosidase inhibition assay, Compounds **4**, **5**, and **10** displayed the strong anti-glucosidase efficacy, with 77.534 to 154.363-fold greater than that of the positive control (acarbose), Compound **3** showed activity with a 0.918-fold change, and the remaining compounds exhibited fewer activities (lower than a 0.663-fold change). Therefore, molecular docking studies were done to examine the interactions among α-glucosidase with active substances. Compound **5** had a lower free binding energy (−9.1 kcal/mol) than Compounds **10** (−8.7 kcal/mol), **4** (−8.9 kcal/mol), and **3** (−5.0 kcal/mol) according to the molecular docking results. The three Compounds **4**, **5**, and **10** had lower free binding energies than acarbose (−7.9 kcal/mol), indicating that the lower the free binding energy, the better the compound’s affinity for the targeted receptor. As a result, the findings show that the active molecules bind to glucosidase more readily than acarbose (Figure 4). These findings are consistent with those acquired during an in vitro study. A detailed analysis of the pose of the docking is presented in Table 3.

**Figure 5:**
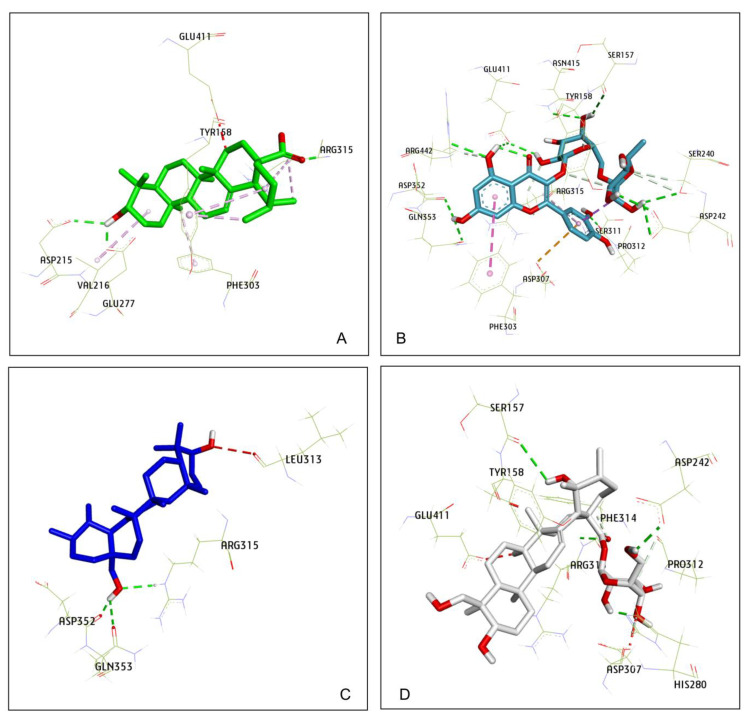
Compounds docked to the binding pocket of α-glucosidase. (**A**) Compound **5**—most active; (**B**) Compound **10**; (**C**) Compound **4**; (**D**) Compound **3**—least active.

The hydroxyl group (C_3_-OH) of **5** was located in the hydrophobic pocket, surrounded by the residues of Asp215 and Glu277, which form stable polar bonds (Figure 5A). Therefore, the active site was not occupied by water molecules. Before binding to the inhibitor, these water molecules catalyze the hydrolysis of the enzyme in the presence of glucose. Water molecules are also responsible for bridging the carboxylate groups of the catalytic Glu and Asp residues and participate in hydrolysis. The other water molecules are thought to form a water reservoir and provide water for subsequent hydrolytic events. Thus, the surrounding environment is primarily hydrophobic, which helps increase their mobility. These details were all mentioned in the study of Yamamoto et al. on the basis of the isomaltase structure from *S. cerevisiae* [28]. Similarly, the hydroxyl groups of **4** (C_28_-OH) and **10** (C_7_-OH) form stable bonds with Asp352, helping them to not be displaced by water molecules (Figure 5B,C). However, in the structure of Compound **3**, the hydroxyl groups could not create polar interactions with the amino acids in the hydrophobic pocket (Figure 5D), thus suggesting that this compound could inhibit the function of the targeted enzymes at a higher concentration than the reference ligand (acarbose), which is consistent with the results of the in vitro anti-α-glucosidase assay.

Another important hydrogen bond interaction was observed among the studied compounds and Tyr158, His280, and loop 310–315, which are located at the entrance of the active site pocket [28]. A detailed analysis showed that Compound **3** was involved in several H–bond interactions with Ser157, Tyr158, Asp242, His280, Asp307, Pro312, Phe314, Arg315, and Glu411. The residues of Arg315 and Gln353 were the main interactions between **4** and α-glucosidase. Compound **5** created two pi–alkyl interactions with Tyr158 and Phe303, and one alkyl with Val216, which is different from **4**, suggesting this interaction might lead to an enhancement in the inhibition activity of this compound. Compound **10** formed one pi–pi stacking (Phe303), one pi–anion (Asp307), and some H-bonds in the interactions with Ser157, Ser240, Asp242, Phe314 Ser311, Agr315, Gln353, Glu411, and Arg442 (Figure 5 and Table 3).

**Figure 6:**
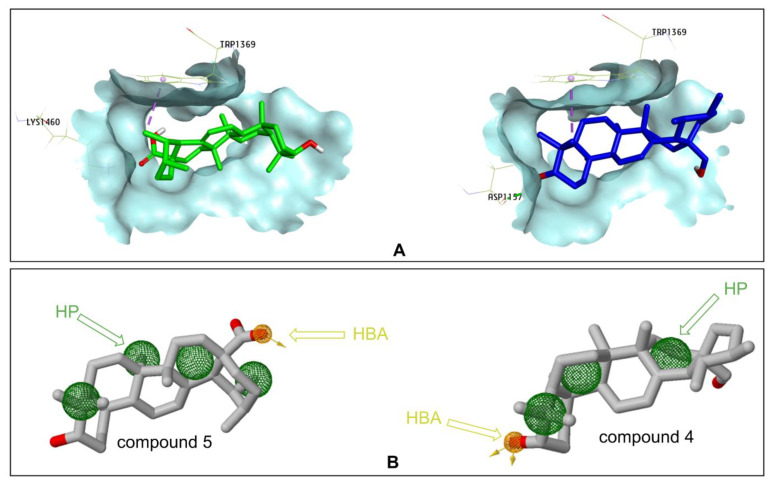
(**A**) Compound **4** (blue) and Compound **5** (green) in the binding site of human intestinal α-glucosidase; (**B**) pharmacophore model. HBA: Hydrogen bond acceptors are depicted as yellow-brown arrows; HP: hydrophobic areas are depicted as green spheres.

Compounds **4** and **5** were studied for the inhibition of human intestinal α-glucosidase. The molecular docking results show that Compound **4** had a lower free binding energy (−9.0 kcal/mol) than Compound **5** (−7.4 kcal/mol). Two important interactions were observed between Compound 4 and the human intestinal α-glucosidase enzyme; the side chains of Asp1157 formed a hydrogen bond with the C_3_-OH group, and C_23_ created a pi–sigma interaction with Trp1369. These interactions were also observed with acarbose in the study of Ren et al. [29]. An interaction with Trp1369 was only observed in Compound **5** at C_29_, and C_28_ (the acid group (C_17_-COOH)) formed a hydrogen bond with Lys1460. Lys1460 acts as a base because it accepts protons from the acid group of **5** (Figure 6A).

The pharmacophore models on the interaction with human intestinal α-glucosidase enzyme were generated using ZINCPharmer online [30]. It was also revealed that there were four hydrophobic areas (HPs) and one hydrogen bond acceptor (HBA) in Compound **5**, and the hydrogen bond acceptor was presented only on C_17_-COOH. Compound **4** had three HPs and one HBA. The hydrogen bond acceptor was seen on C_28_-OH of Compound **4** (Figure 6B).

## 3. Discussion

The roots of *M. speciosa* have been known as traditional medicine materials and used for the treatment of joint pain, menoxenia, blood deficiency sallow, rheumatoid arthritis, amenorrhea, hepatitis, tuberculosis, and chronic bronchitis [15]. Several flavonoids and isoflavones, such as flavonoids naringenin, liquiritigenin, garbanzol, calycosin, and isoflavones 2′-hydroxybiochanin A, 7-hydroxy-6,4′-dimethoxyisoflavone, 2′,5′,7-trihydroxy-4′-methoxyisoflavone, and 6-methoxycalopogonium isoflavones A, were reported to occur in the roots of this plant [8]. The polysaccharide fraction MSCP2 (molecular weight of 2.85 × 10^4^ Da), composed of fucose, arabinose, galactose, glucose, and xylose, was found to possess immunomodulatory properties due to an enhancement in its pinocytic capacity and the levels of NO and cytokines in RAW 264.7 cells in vitro [14]. The ethanol extract of this material was observed to contain medicarpin and maackiain, two known pterocarpans that inhibited leukotriene secretion from RBL-2H3 cells and were toxic to HL-60 leukemia cells [18]. Two rotenoids, millettiaosas A–B, were isolated from the roots of *M. speciosa* and found to have moderate cytotoxicity against MCF-7, HCT-116, A549, and HepG-2 cell lines, with IC_50_ values ranging from 10 to 26 µM in vitro [31]. In the present study, ten isolated compounds from this plant were identified to be friedelin, rotundic acid, pedunculoside, uvaol, ursolic acid, gypenoside XVII, pterocarpin, syringin, daidzin, and rutin. It is worth noting that the ursane-type triterpenes **4** and **5**, gypenoside XVII (**6**), and pterocarpin (**7**) were identified for the first time in the roots of this species and displayed a remarkable inhibition for NO production. Ursolic acid was found to strongly suppress the NO production in lipopolysaccharide (LPS)-stimulated RAW 264.7 cells; however, it also caused moderate cytotoxicity against the cells. Ursolic acid has been known for its anti-cancer activity and multifunction effect on tumorigenesis, cell differentiation, and anti-angiogenic effect [32,33]. In a previous report by Kim et al., ursolic acid isolated from *Phryma leptostachya* var. *asiatica* was found to be effective on NO formation by 80.6% at a concentration of 40 µg/mL [34]. Even though Compound **5** (ursolic acid) seemed to be the best inhibitor for NO production in this study, it also caused cytotoxicity against RAW 264.7 macrophage cells at concentrations higher than 100 µg/mL. As a result, Compound **6** (IC_50_ = 93.9 µg/mL) was likely a more potent inhibitor and showed no cytotoxicity in comparison to **5** in the search of a promising candidate for anti-inflammatory drug development. 

Among the four ursane-type triterpenes, Compounds **2**–**4** suppressed the NO production in RAW 264.7 macrophage cells without cytotoxicity and also displayed better inhibition compared to that of Compound **1**, an oleanane-type triterpene occurring in the roots of *M. speciosa*.

The phenolic Compounds **7**–**10** were found in the extract of the roots of this plant; however, it seems that their inhibitory activity on NO production was less remarkable than those of ursane-type triterpenes (Table 1). Among the phenolic compounds, pterocarpin (**7**) showed an IC_50_ of 228.90 µg/mL and was, for the first time, found to be active against NO production. In contrast, Compounds **8** (syringin) and **10** (rutin) displayed insignificant effects on NO production in RAW 264.7 cells; the results are in the line with the discovery of [35], who found NO production was not blocked by syringin even at a high concentration of 1000 µM. This compound was described as an immunomodulator exerting an anti-allergic effect rather than an anti-inflammatory effect. In addition, rutin was also found to mediate the NO synthesis in human umbilical vein endothelial cells by inducing eNOS mRNA expression, protein synthesis, and eNOS activity [36].

Diabetes is a metabolic epidemic disease and is the third cause of death for humans after cancer and cardiovascular disease. Enzyme α-glucosidase is located on the epithelium of the small intestine and breaks down the ingested disaccharides into glucose. The inhibitors of α-glucosidase inhibit the breakdown of starchy foods; this causes the suppression of postprandial hyperglycemia in the human body. Therefore, α-glucosidase inhibitors have often been investigated and developed into drugs for type 2 diabetes treatments. 

With regard to the anti-α-glucosidase activity, it seems that the test compounds and the positive control acarbose fall in three groups. Group I consists of three strong active compounds, **4**, **5**, and **10**, the activities of which were about 70 times higher than that of acarbose. Compound **3** and acarbose are classified into Group II, where Compound **3** moderately inhibits α-glucosidase with a change of 0.918-fold compared to acarbose. Group III consists of Compounds **1**, **2**, and **6**–**9,** which showed no or poor anti-glucosidase efficacy. Compounds **4**, **5**, and **10** showed the best inhibition with IC_50_ values much lower than that of acarbose (2.0, 1.1, and 2.2 versus 169.8 µg/mL, respectively). The anti-α-glucosidase activity of rutin (**10**) was described by [37]; in their work, α-glucosidase inhibition by rutin (a purchased sample) varied in the range of 10.6–52.6% at tested concentrations of 50–250 µg/mL.

In addition, uvaol (**4**) and ursolic acid (**5**) are ursane-type triterpenes, and they were observed as the most potent inhibitors for α-glucosidase. In the work, ursolic acid (**5**) showed the strongest α-glucosidase inhibition; it showed an IC_50_ of 1.1 µg/mL and completely inhibited this enzyme (91.5%) at 4 µg/mL. These results are consistent with the data previously reported by Ding et al., in which oleanolic acid and ursolic acid possessed IC_50_ values of (6.35 ± 0.02) × 10^−6^ and (1.69 ± 0.03) × 10^−5^ mol/L (equivalent to 7.71 µg/mL of ursolic acid), respectively, and ursolic acid inhibited α-glucosidase in a non-competitive manner [38]. Zhang et al. also described the effectiveness of α-glucosidase inhibition by pentacyclic triterpenes in the order of ursolic acid, corosolic acid, bentulinic acid, and oleanolic acid; ursolic acid was found to the best inhibitor, with an IC_50_ of 12.1 µM (equivalent to 5.51 µg/mL of ursolic acid) [39]. The extracts of 14 *Salvia species*, which contain ursolic acid and oleanolic acid as primary constituents, were found to have a substantial inhibitory effect on α-glucosidase, with IC_50_ values ranging from 17.6 to 173.0 μg/mL [40]. Uvaol and ursolic acid have the same skeleton of ursane-type triterpenoid and they differ from the substitutive group of C-28, where uvaol is in the CH_2_OH group and ursolic acid is in the COOH group (Figure 1). Ursolic acid and uvaol isolated from *Clinopodium taxifolium* showed α-glucosidase inhibition, with IC_50_ values of 72.7 and 521.0 µg/mL, respectively [41]. According to Wang et al., ursolic acid (a commercial sample) and acarbose were tested against α-glucosidase, and their IC_50_ values were determined to be 213 µg/mL for ursolic acid and 1160 µg/mL for acarbose [42]. In our study, the α-glucosidase inhibitory activities of **2** and **3** derived from the roots of *M. speciose* were reported and evaluated for the first time. Interestingly, Compound **3** (pedunculoside) is also known as a ursane-type triterpenoid, but it showed moderate α-glucosidase inhibitory activity (Table 2). Similar to the chemical structure of uvaol, Compounds **2** and **3** are derivatives of ursolic acid, with hydroxyl groups linked to C_23_ and C_19_; however, Compound **3** also has an ester linkage at C_28_ with glucose. The difference in the structures of **2** and **3** may result in a reduction in α-glucosidase enzymatic activities compared to those of **4** and **5** (Figure 1 and Table 3). Therefore, in this study, we also approached molecular docking to predict the orientation of Compound **3** and Group I at the active site of the α-glucosidase enzyme. The main purpose was to clarify the mechanism and further strengthen our argument outlined above. 

Molecular docking was utilized to predict the binding pose of the studied compounds in the active site of α-glucosidase. Through molecular docking analysis with Autodock Vina, four compounds, **3**–**5** and **10**, were found to insert into the hydrophobic pocket of α-glucosidase and were surrounded by many polar amino acids. Ursolic acid (**5**) was found to mainly interact with six amino acid residues (Tyr158, Asp215, Val216, Glu277, Phe303, and Arg315). Three amino acids (Arg315, Asp352, and Gln353) formed H-bonds with uvaol (**4**), and the interactions were observed among the rutin (**10**) and the twelve residues. Compound **3** could not create polar interactions with the key amino acids deep at the bottom of the hydrophobic pocket as the above compounds. Therefore, the active site is still occupied by water molecules, so the hydrolytic process can occur at the beginning. However, Compound **3** shielded the entrance to the bag and prevented the supply of water for subsequent hydrolysis events. This may be the reason Compound **3** was less active than the other compounds. Interestingly, further research into the binding energies of two compounds to inhibit human intestinal α-glucosidase revealed that Compound **4** had a lower free binding energy than Compound **5**. Pharmacophore models on the interaction with human intestinal α-glucosidase were generated using ZINCPharmer; the generated pharmacophore models could assist medicinal chemists in designing inhibitors against α-glucosidase based on the structures of these two compounds. From the pharmacophore models, it is predicted that difference substituents at C_17_ play the most important role in differentiating the activities of two compounds. Any systematic variations of substituents that change HBA and HP interactions can help in the discovery of molecules, with better biological effects than Compound **5** in the binding site of α-glucosidase. 

The present results in the molecular docking study of the ursane-type triterpenes show similarities with previous studies on α-glucosidase targeting [37,43,44,45]. According to Dubey et al., a docking study of rutin was visualized by Discovery Studio, in which rutin demonstrated an inhibition constant of 67.62 µm and binding energy of −7.01 kcal/mol with α-glucosidase (PDB ID: 3A4A) by non-covalent interaction [37]. 

Almost all of the isolated substances from the roots of *M. speciosa* exhibited low AChE inhibition, except Compound **5** showed moderate activity. In general, AChE inhibitors enhance cholinergic neurotransmission; therefore, the known phytochemicals of low toxicity would be safe for use in traditional medicine. 

## 4. Materials and Methods

### 4.1. Plant Materials

The roots of *M. speciosa* (Figure S1—Supplementary Materials) were collected from October 2019 in Pumat National Park, Nghean province, Vietnam. The plant materials were identified by Dr. Quoc Binh Nguyen, Vietnam National Museum of Nature, Vietnam Academy of Science and Technology, Hanoi, Vietnam. The voucher samples (No. MS-102019) were deposited in the same museum.

### 4.2. General Procedures

Electron-spray ionization-mass spectrometry (ESI-MS) spectra were measured on an Agilent 1100 LC-MSD-Trap-SL system (Agilent Technologies, Santa Clara, CA, USA). The Bruker Avance 500 NMR spectrometer was used to record ^1^H-NMR, ^13^C-NMR, and DEPT spectra in CDCl_3_. Tetramethylsilane (TMS) served as an internal standard, and the chemical shifts were measured in parts per million (ppm) in comparison to the standard.

The α-Glucosidase enzyme (CAS 9001-42-7) from *Saccharomyces cerevisiae*, *p*-nitrophenyl-*α*-D-glucopyranoside (CAS 3767-28-0), 4-nitrophenol (CAS 100-02-7), and dimethyl sulfoxide (DMSO, CAS 67-68-5) were purchased from Sigma-Aldrich (Burlington, MA, USA). Silica gel (40–63 µm, 60 Å, Merck, Darmstadt, Germany) was employed to use for open column chromatography (CC). Silica gel thin-layer chromatography (TLC) that was coated onto F_254_ aluminum plates was used for monitoring column separation and analyzing the purity of the isolated compounds.

### 4.3. Isolation and Characterization of Phytochemical Constituents

The roots of *Millettia speciosa* (5.3 kg) were extracted with ethanol at 60 °C (10 L × 3) by ultrasound-assisted extraction (UAE), and total ethanol extract was evaporated under reduced pressure to yield the ethanol crude extract (575 g). Then, it was suspended in water and partitioned successively with *n*-hexane, ethyl acetate, and butanol to afford *n*-hexane extract (MSH-61 g), ethyl acetate extract (MSE-129 g), butanol extract (MSB-143 g), and a water-soluble fraction (MSW-121 g), respectively. 

The MSH (61 g) was isolated by silica gel column chromatography (CC) (150 g, 150 cm × 10 cm) to collect five fractions (Frs. MSH1-MSH5). Fraction MSH5 was re-chromatographed by CC (80 g, 80 × 1.5 cm) eluted with a gradient of hexane–ethyl acetate (15/1; 9/1; *v*/*v*) to yield friedelin (**1**) (16.5 mg).

The MSE (129 g) was applied to CC (300 g, 150 cm × 10 cm); then it was eluted with a mixture of chloroform–methanol with gradient (100/0, 20/1, 10/1, 5/1, 2/1, 1/1) to afford ten fractions (Frs. MSE1-MSE10). Fraction MSE2 (20.6 g) was separated by CC (80 g, 80 × 1.5 cm) and eluted with a mixture of hexane–ethyl acetate (3/7; *v*/*v*) to afford ursolic acid (**2)** (12.8 mg). Fraction MSE3 (18.3 g) was separated by CC (300 g, 80 × 3 cm) and eluted with a mixture of n-hexane–acetone (15/1; *v*/*v*) to collect 3 fractions (MSE3.1-MSE3.3). The MSE3.2 was re-chromatographed by CC (140 g, 80 × 1.5 cm) with a gradient of hexane–ethyl acetate (10/1; 7/1; *v*/*v*) to yield rotundic acid (**3**) (14.2 mg). MSE3.3 was separated by CC (50 g, 80 cm × 1.5 cm) and eluted with n-hexane–ethyl acetate (15:1) to collect uvaol (**4**) (14.5 mg). Fraction MSE4 (16.2 g) was subjected to column chromatography and eluted with n-hexane–ethyl acetate (15:1) to afford pterocarpin (**7**) (21.5 mg). Fraction MSE6 (21.3 g) was subjected to CC (100 g, 80 × 1.5 cm) to collect (3*β*,4*α*)-3,19,23-trihydroxy-urs-12-en-28-oic acid *β*-D-glucopyranosyl ester (**5**) (10.2 mg).

The MSB (143 g) was applied to column chromatography on silica gel (150 cm × 10 cm) and eluted with a CHCl_3_–MeOH mixture with a step gradient of MeOH to increase the polarity (20:1; 10:1; 7:1; 5:1; 2:1; *v*/*v*) to collect 8 fractions (Frs. MSB1-MSB8). Fraction MSB2 (2.9 g) was subjected to silica gel column chromatography (300 g, 60 × 3 cm) and eluted with a mixture of chloroform and methanol (15:1, 8:1) to yield sinapyl alcohol 4-O-glucoside (**8**) (52.0 mg). Fraction MSB3 was resolved by first using a Sephadex LH-20 column eluted with CHCl_3_–MeOH (1:1) and then a silica gel column eluted with a mixture of CHCl_3_–MeOH (20:1 to 5:1) to obtain five minor fractions (MS3.1 to MSB3.5). The MSB3.4 was applied to silica gel column chromatography (CHCl_3_: MeOH = 20:1) and then thin-layer chromatography with (CHCl_3_–MeOH = 20:1) to produce the yellow powder denoted as daidzein-7-O-*β*-D-glucopyranoside (**9**) (23.0 mg). Fraction MSB4 was subjected to the preparative thin-layer chromatography (pTLC) (typical plate dimensions: 20 cm × 20 cm, 2.5 mm SiO_2_ thickness) and eluted with a mixture of chloroform and methanol (8:1, 6:1) to produce rutin (**10**) (57.0 mg). The purification of fraction MSB8 (12.6 g) was performed using an RP-18 column eluted with MeOH–H_2_O (45:55; *v*/*v*) to obtain four minor fractions (Frs. MSB8.1–MSB8.4). The minor fraction MSB8.2 (2.7 g) was isolated by preparative HPLC (ZORBAX SB-C18 (5 μm, 21.2 × 100 mm) with MeOH:–H_2_O (55:45) for 10 min (10 mL min^−1^) to afford gypenoside XVII (**6**) (6.2 mg).

### 4.4. Structural Characterization of the Isolated Compounds 

The MS and NMR data of Compounds **1**–**10** isolated from the roots of *Millettia speciosa* are presented in Data S1 in the Supplementary Materials.

### 4.5. In Vitro Evaluation of NO Production Inhibitory Activity of the Isolated Compounds

The RAW 264.7 macrophage cell line was obtained from the Institute of Biology of the Vietnam Academy of Science and Technology and cultured in Dulbecco’s Modified Eagle Medium (DMEM, Gibco, Thermo Fisher Scientific, Waltham, MA, USA) containing 2 mM of L-glutamine, 10 mM of HEPES, and 1 mM of sodium pyruvate. It was supplemented with 10% fetal bovine serum (FBS) and incubated at 37 °C in humidified air with 5% CO_2_. RAW 264.7 macrophage cells in DMEM medium in 96-well plates were incubated for 24 h, and NO production was stimulated by LPS (1 μg/mL). Next, 100 μL of Griess reagent (50 μL of 1% (*w*/*v*) sulfanilamide in 5% (*v*/*v*) phosphoric acid and 50 μL of 0.1% (*w*/*v*) *N*-1-naphthylethylenediamine dihydrochloride) was added, and it was incubated at room temperature for about 10 min. The Griess Reagent System from Promega Cooperation (USA) was used to determine the presence of nitrite. The microplate reader was used to assess absorption at 540 nm. N^G^-Methyl-L-arginine acetate (L-NMMA) (Sigma) was used as a positive control at doses of 100, 20, 4, and 0.8 g/mL. The IC_50_ values were calculated from non-linear regression analysis based on the dose–response curves using TableCurve 2Dv4 software. The experiments were repeated at least three times independently.

### 4.6. Cell Viability Assay for the Evaluation of the Cytotoxicity of the Isolated Compounds

The test compounds were diluted and added to 96-well microtiter plates with concentrations similar to those of the NO assay. The cells were diluted to a suitable cell density. One hundred eighty microliters of the cells were added to each well of the plate and incubated at 37 °C, 5% CO_2_ for 72 h. After the incubation, 10 µL of MTT solution (5 mg/mL) was added. After 4 h, the MTT was removed and the formazan crystals were dissolved in 50 µL of 100% DMSO. The OD was read by a microtiter plate reader at a wavelength of 540 nm. Cell viability was calculated based on the formula as follows:

Cell viability (%) = [(OD Sample)/(OD Control)] × 100%,

where OD is the optical density recorded at 540 nm.

### 4.7. In Vitro Bioassay for α-Glucosidase Inhibition of the Isolated Compounds from the Roots of Milletia speciosa 

The isolated compounds were tested for their α-glucosidase inhibitory activity using the method previously reported by Ting et al. (2005) [46]. The test compounds were dissolved in dimethylsulfoxide (DMSO) to form stock solutions. *p*-NPG (*p*-nitrophenyl α-D-glucopyranoside) (Sigma-Aldrich) and 0.2 U/mL of α-glucosidase from *Saccharomyces cerevisiae* (Sigma-Aldrich) were prepared in 100 mM of potassium phosphate buffer with a pH of 6.8. UV absorption at 410 nm was measured using the BIOTEK machines. The half-maximal inhibitory concentration (IC_50_) values were calculated from non-linear regression analysis based on the dose–response curves.

### 4.8. In Vitro Bioassay for Acetylcholinesterase Inhibition of the Isolated Compounds 

The acetylcholinesterase activity was determined by a colorimetric assay based on Ellman’s methodology. The test compounds were prepared in a series of concentrations from 1 to 256 µg/mL. They were dissolved in DMSO and then diluted with buffer (50 mM of Tris-HCl, with a pH of 8 containing 0.1 M NaCl), 0.1% bovine serum albumin (BSA), 25 µL of acetylthiocholine iodide. The plate was incubated at 25 °C for 15 min. The yellow 5-thio-2-nitrobenzoate anion was formed and could be detected at 405 nm. Each assay was done in triplicate. The percentage of inhibition of AChE was determined by the following formula:

Inhibition (%) = [(OD of control − OD of sample)/OD of control] × 100.

### 4.9. Molecular Docking Study for Anti-α-Glucosidase Inhibition

Since the crystallographic structure of *Saccharomyces cerevisiae* α-glucosidase enzyme is not available in the Protein Data Bank (PDB), the three-dimensional structure of α-glucosidase was built using homology modeling on the Swiss-Model website (https://swissmodel.expasy.org/, accessed on 10 September 2021). The template structure was searched on NCBI protein BLAST to model the protein of interest. Swiss-Model suggested a crystal structure of isomaltase enzyme from *S. cerevisiae* (PDB ID: 3AJ7) with 72.4% identity and 91% query coverage [28]. The stereochemical aspects of the model were inspected by checking the Ramachandran plot (see Supplementary Materials); it can be considered a liable model for further docking studies. This homology modeling was used to investigate the interactions of compounds with the active site of α-glucosidase [43,44,47]. The crystal structure of human intestinal α-glucosidase in a complex with acarbose inhibitor (PDB ID: 3TOP) was retrieved from the PDB. The three-dimensional structures of the selected compounds were created by Gaussview, and the energy minimization was carried out in Gaussian [48]. AutoDock Vina was employed to set up and perform the docking calculations by the PyRx program [49,50]. In this study, we performed the docking study assuming a rigid protein and considering the conformational space of the ligands to analyze the inductive effect of the hybrid compounds. In the docking analysis, the binding site was enclosed in a box with the number of grid points in x × y × z dimensions (25 Å × 25 Å × 25 Å). The center of the grid box was placed at x  =  22.2262, y  =  −8.1477, z = 23.9431 for *Saccharomyces cerevisiae* α-glucosidase enzyme and x  =  22.2262, y  =  −8.1477, z = 23.9431 for the human intestinal α-glucosidase. The outputs of the AutoDock Vina modeling studies were analyzed using Discovery Studio Visualizer.

### 4.10. Statistical Analysis

The assays were performed at least in triplicate and the values are expressed as the mean ± SD (standard deviation). The half-maximal inhibitory concentration (IC_50_) values were calculated from non-linear regression analysis based on the dose–response curves.

## 5. Conclusions

Ursane-type triterpenes **4** and **5**, gypenoside XVII (**6**), and pterocarpin (**7**) were isolated and identified from the roots of *M. speciosa* for the first time. The isolated ursane-type triterpenes **2**–**5** showed a remarkable inhibition for NO production in LPS-stimulated RAW 264.7 cells. In addition, Compounds **3** and acarbose inhibited α-glucosidase at a similar potential level. Compounds **4**, **5**, and **10** showed the best α-glucosidase inhibition, with IC_50_ values ranging from 1.1 to 2.2 µg/mL. Through the molecular docking study of these inhibitors with α-glucosidase, the interactions of **3**–**5** and **10** at the active site pocket were observed and provided an explanation for the in vitro results. These data suggest that the phytochemicals derived from the roots of *M. speciosa* may be promising lead molecules for further studies on the development of anti-inflammation and anti-diabetes drugs.

## Figures and Tables

**Table 1:** Half-maximal inhibitory concentration (IC_50_) for the inhibition of NO production by the isolated compounds from *Millettia speciosa*.

Compound ^1^	IC_50_ (µg/mL) ^2^
**1**	>500
**2**	241.3 ± 8.2
**3**	273.1 ± 8.2
**4**	246.5 ± 18.7
**5**	43.9 ± 3.7
**6**	93.9 ± 5.4
**7**	228.9 ± 18.6
**8**	303.1 ± 11.0
**9**	>500
**10**	449.5 ± 5.2
PC	8.6 ± 0.9

^1^ Compounds—**1**: friedelin; **2**: rotundic acid; **3**: pedunculoside; **4**: uvaol; **5**: ursolic acid; **6**: gypenoside XVII; **7**: pterocarpin; **8**: syringin; **9**: daidzin; **10**: rutin. PC: positive control with L-NMMA. ^2^ Values are the means of three replicates  ±  standard deviation (SD).

**Table 2:** Half-maximal inhibitory concentration (IC_50_) for inhibition of α-glucosidase by the isolated compounds from *Millettia speciosa*.

Compound ^2^	IC_50_ (µg/mL) ^1^	Fold Change
**1**	>256	<0.663
**2**	>256	<0.663
**3**	184.9 ± 10.05	0.918
**4**	1.96 ± 0.09	86.632
**5**	1.1 ± 0.05	154.363
**6**	>256	<0.663
**7**	>256	<0.663
**8**	>256	<0.663
**9**	>256	<0.663
**10**	2.2 ± 0.09	77.534
Positive control ^3^	169.8 ± 7.05	1.000

^1^ Values are the means of three replicates  ±  standard deviation (SD). ^2^ Compounds—**1**: friedelin; **2**: rotundic acid; **3**: pedunculoside; **4**: uvaol; **5**: ursolic acid; **6**: gypenoside XVII; **7**: pterocarpin; **8**: syringin; **9**: daidzin; **10**: rutin. ^3^ Acarbose was used as a positive control in the evaluation of α-glucosidase inhibition activity.

**Table 3:** Interaction residues of compounds obtained from molecular docking simulation.

Compound ^1^	Hydrogen Bond Interacting Residues ^2^
**3**	Ser157, Tyr158 (unfavorable bump), Asp242, His280, Asp307 (unfavorable bump), Pro312, Phe314, Arg315, Glu411 (unfavorable bump).
**4**	Leu313 (unfavorable bump), Arg315, Asp352, Gln353.
**5**	Tyr158 (pi–alkyl), Asp215, Val216 (alkyl), Glu277, Phe303 (pi–alkyl), Arg315, Glu411 (unfavorable bump).
**10**	Ser157, Ser240, Asp242, Phe303 (pi–pi stacked), Asp307 (pi–anion), Phe314, Ser311, Agr315, Asp352, Gln353, Glu411, Arg442.
Acarbose	Asp69, Asp215, Ser240, Asp242, His280, Phe303, Pro312, Arg315 (unfavorable bump), Arg442 (unfavorable bump).

^1^ Compounds—**3**: pedunculoside; **4**: uvaol; **5**: ursolic acid; **10**: rutin; acarbose: control. ^2^ Ser: serine; Tyr: tyrosine; Asp: aspartic acid; His: histidine; Pro: proline; Phe: phenylalanine; Glu: glutamic acid; Arg: arginine; Val: valine; Gln: glutamine; Leu: leucine.

## Data Availability

The data used to support the findings of this study are included in the article.

## Supplementary Materials

The following are available online at https://www.mdpi.com/article/10.3390/plants11030388/s1, Data S1: MS and NMR data of Compounds **1**–**10** isolated from *Millettia speciosa*. Figure S1: Image of the roots of *Millettia speciosa*, Figure S2: Isolation scheme of isolated Compounds **1**–**10** from the fruits of *Millettia speciosa*, Figure S3: Ramachandran plot analysis of the structure of *Saccharomyces cerevisiae* α-glucosidase model, Figure S4: 2D binding model of Compound **3** in the active site of *Saccharomyces cerevisiae* α-glucosidase enzyme; Figure S5: 2D binding model of Compound **4** in the active site of *Saccharomyces cerevisiae* α-glucosidase enzyme; Figure S6: 2D binding model of Compound **5** in the active site of *Saccharomyces cerevisiae* α-glucosidase enzyme; Figure S7: 2D binding model of Compound **10** in the active site of *Saccharomyces cerevisiae* α-glucosidase enzyme; Figure S8: 2D binding model of Compound **4** in the active site of human intestinal α-glucosidase enzyme; Figure S9: 2D binding model of Compound **5** in the active site of human intestinal α-glucosidase enzyme. Table S1: α-Glucosidase inhibition of by the isolated compounds from *Millettia speciosa*. Table S2: Half-maximal inhibitory concentration (IC_50_) values for acetylcholinesterase inhibition by the isolated compounds from *Millettia speciose.*
