# Supplementary material for: Inhibition of α-Glucosidase, Acetylcholinesterase, and Nitric Oxide Production by Phytochemicals Isolated from Millettia speciosa—In Vitro and Molecular Docking Studies
Source: Plants (Basel). 2022 Jan 30;11(3):388. doi: 10.3390/plants11030388 (PMC8840612; doi:10.3390/plants11030388)
Supplement: Supplementary file 1 [file plants-11-00388-s001.zip › plants-1524197-supplementary.pdf]

Supplementary Information

Inhibition of *α*-Glucosidase, Acetylcholinesterase and Nitric Oxide Production by Phytochemicals Isolated from *Millettia speciosa*: *In vitro* and Molecular Docking Studies

Nguyen Ngoc Tuan[1], Huong Nguyen Thi[2,3], Chau Le Thi My[4], Tang Xuan Hai[5], Hieu Tran Trung[2], Anh Nguyen Thi Kim[1], Thanh Nguyen Tan[4], Tan Le Van[6], Cuong Quoc Nguyen[7], Quang De Tran[7], Ping-Chung Kuo[8], Quang Le Dang[9,10,*], Tran Dinh Thang[1,*]

[1] Institute of Biotechnology and Food Technology, Industrial University of Ho Chi Minh City, Ho Chi Minh, 71408, Vietnam; nguyenngoctuan@iuh.edu.vn (N.N.T.), nguyenthikimanh@iuh.edu.vn (A.N.T.K.), thangtd@iuh.edu.vn (T.D.T.)

[2] School of Natural Sciences Education, Vinh University, Nghean, 43100, Vietnam; nguyenthihuongtn@hdu.edu.vn (H.N.T.), trantrunghieu94tc@gmail.com (H.T.T.)

[3] Faculty of Natural Sciences, Hong Duc University, Thanh Hoa, Vietnam; nguyenthihuongtn@hdu.edu.vn (H.N.T.)

[4] School of Chemistry, Biology and Environment, Vinh University, Nghean, 43100, Vietnam; lemychau83@gmail.com (C.L.T.M.), nguyentanthanhvn@gmail.com (T.N.T.)

[5] Nghe An Obstetric & Paediatric Hospital, Vinh City, Vietnam; bstangxuanhai@gmail.com (T.X.H.)

[6] Faculty of Chemical Engineering, Industrial University of Ho Chi Minh City, Ho Chi Minh, 71408, Vietnam; levantan@iuh.edu.vn (T.L.V)

[7] Department of Chemistry, College of Natural Sciences, Can Tho University, Can Tho 900000, Vietnam; ncquoc99@gmail.com (C.Q.N.), tqde@ctu.edu.vn (Q.D.T.)

[8] School of Pharmacy, College of Medicine, National Cheng Kung University, Tainan 70101, Taiwan; z10502016@ncku.edu.tw (P.-C.K.)

[9] R&D Center of Bioactive Compounds, Vietnam Institute of Industrial Chemistry, Hanoi, 10000, Vietnam; ledangquang2011@gmail.com (Q.L.D.)

[10] Institute for Tropical Technology, Vietnam Academy of Science and Technology, Hanoi, 10000, Vietnam; ledangquang2011@gmail.com (Q.L.D.)

* Correspondence: ledangquang2011@gmail.com (Q.L.D.); thangtd@iuh.edu.vn (T.D.T.)

Key words: *Millettia speciosa*, molecular docking, NO-production, anti-glucosidase, anti-acetylcholinesterase.

S.No.	Contents	Page No.
**1**	Chemistry and spectroscopic data	3-10
**2**	Biological evaluation	11-12
**3**	Detailed description for docking studies	13-21

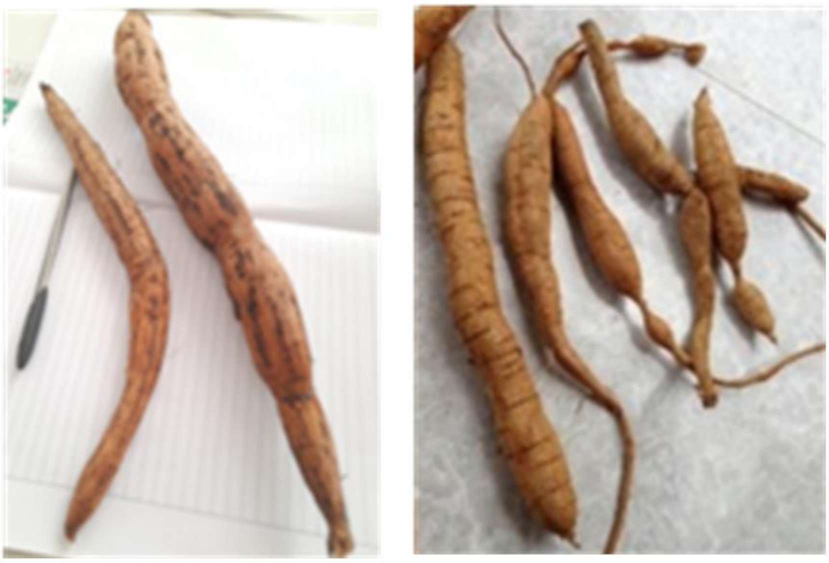

Figure S1. Image of the roots of *Milletia speciosa.*

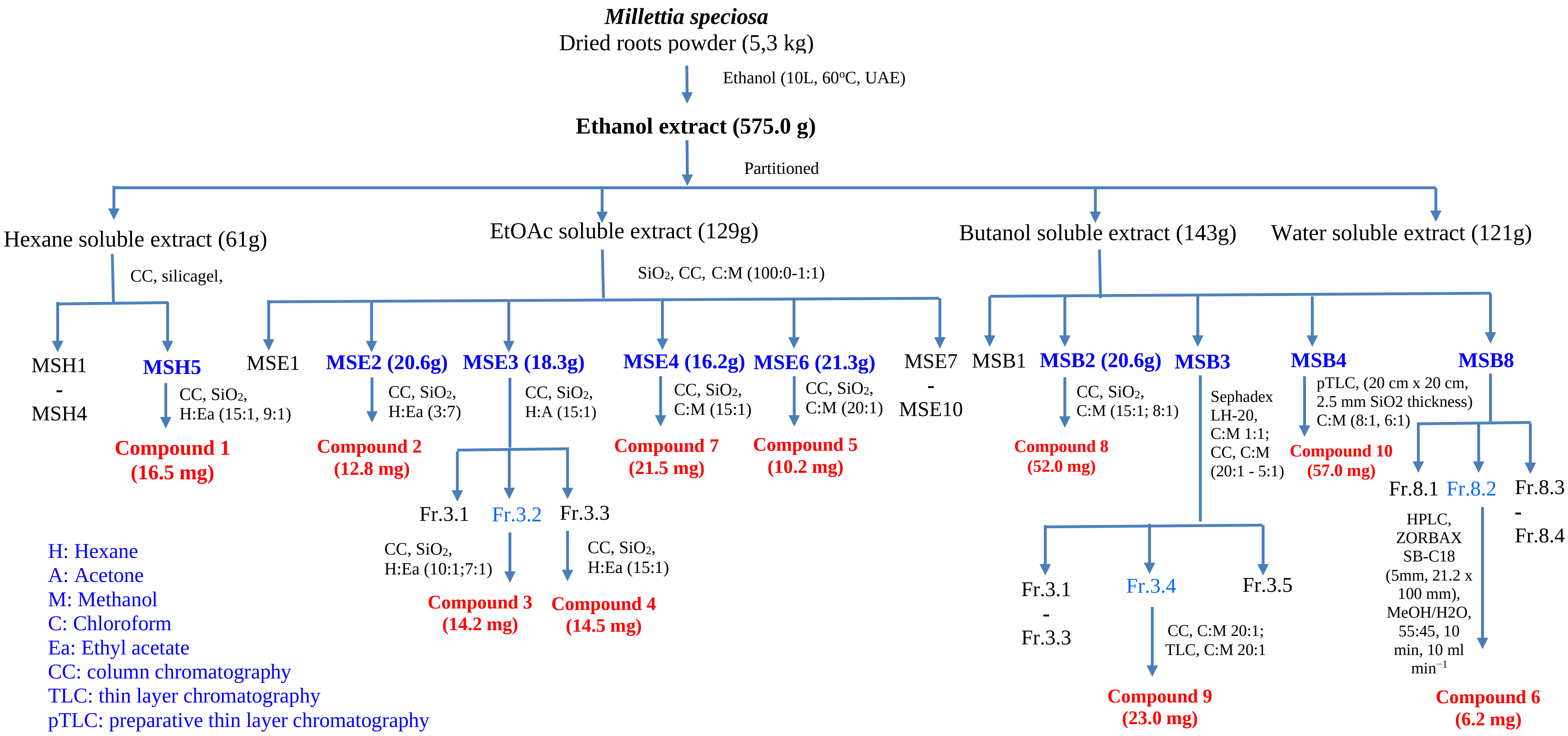

Figure S2. Isolation scheme of compounds **1-10** from *Millettia speciosa.*

Data S1. MS and NMR data of compounds **1-10** isolated from *Millettia speciosa*

Friedelin (1): White powder; m.p. 261 – 262 °C; ESI-MS *m/z*: 427 $[M+H]^+$; ^{1}H-NMR (500 MHz, $CDCl_3$) δ_H (ppm): 2.39 (1H, *dd*, J = 5.0; 3.5 Hz, C_2-H), 2.29 (1H, *m*, C_2-H), 2.25 (1H, *q*, *J* = 7.0 Hz, C_4-H), 1.96 (1H, *ddd*, J = 5.0; 3.0; 3.0 Hz, C_1-H), 1.74 (1H, *d*, *J* = 3.0 Hz, C_6-H), 1.69 (1H, *ddd*, J = 5.5; 5.5; 3.0 Hz, C_1-H), 1.58 (1H, *m*, C_{16}-H), 1.56 (1H, *m*, C_{18}-H), 1.53 (1H, *m*, C_{10}-H), 1.47 (1H, *m*, C_{15}-H), 1.51 (1H, *m*, C_{22}-H), 1.50 (1H, *m*, C_{21}-H), 1.48 (1H, *m*, C_7-H), 1.45 (1H, *m*, C_{11}-H), 1.38 (1H, *m*, C_8-H), 1.37 (1H, *m*, C_{19}-H), 1.36 (1H, *m*, C_7-H), 1.35 (1H, *m*, C_{16}-H), 1.33 (2H, *m*, C_{12}-H), 1.28 (1H, *m*, C_6-H), 1.27 (1H, *m*, C_{15}-H), 1.26 (1H, *m*, C_{11}-H), 1.21 (1H, *m*, C_{19}-H), 1.18 (3H, *s*, C_{28}-H), 1.05 (3H, *s*, C_{27}-H), 1.01 (3H, *s*, C_{26}-H), 1.00 (3H, *s*, C_{29}-H), 0.95 (3H, *s*, C_{30}-H), 0.94 (1H, *m*, C_{22}-H), 0.88 (3H, *d*, J = 6.5 Hz, C_{23}-H), 0.87 (3H, *s*, C_{25}-H), 0.73 (3H, *s*, C_{24}-H); ^{13}C-NMR (125 MHz, $CDCl_3$) δ_C (ppm): 213.2 (C_3), 59.5 (C_{10}), 58.3 (C_4), 53.1 (C_8), 42.8 (C_{18}), 42.2 (C_5), 41.5 (C_2), 41.3 (C_6), 39.7 (C_{13}), 39.3 (C_{22}), 38.3 (C_{14}), 37.5 (C_9), 36.0 (C_{16}), 35.7 (C_{11}), 35.4 (C_{19}), 35.0 (C_{29}), 32.8 (C_{21}), 32.5 (C_{15}), 32.1 (C_{28}), 31.8 (C_{30}), 30.5 (C_{12}), 30.0 (C_{17}), 28.2 (C_{20}), 22.3 (C_1), 20.3 (C_{26}), 18.7 (C_{27}), 18.3 (C_7), 18.0 (C_{25}), 14.7 (C_{24}), 6.8 (C_{23}).

Rotundic acid (2): White amorphous powder, m.p. 271 – 272 °C; ESI-MS *m/z*: 489 $[M+H]^+$; ^{1}H-NMR (500 MHz, DMSO-d_6) δ_H (ppm): 5.16 (1H, *d*, *J* = 3.5 Hz, C-H_{12}), 4.38 (1H, *m*, C_3-H), 4.13 (1H, *s*, C_{23}-H_α), 3.71 (1H, *s*, C_{23}-H_β), 3.09 (1H, *t*, *J* = 7.5; 10.5, C_{18}-H), 1.60 (3H, *s*, C_{30}-H), 1.52 (3H, *s*, C_{27}-H), 1.29 (3H, *s*, C_{29}-H), 1.15 (3H, *s*, C_{25}-H), 0.85 (3H, *s*, C_{26}-H), 0,70 (3H, *s*, C_{24}-H); ^{13}C-NMR (125 MHz, DMSO-d_6) δ_C (ppm): 182.3 (C_{28}), 140.0 (C_{13}), 129.5 (C_{12}), 74.2(C_3), 73.6 (C_{19}), 67.6 (C_{23}), 55.1 (C_{18}), 48.9 (C_5), 49.1 (C_{17}), 48.5 (C_9), 43.3 (C_4), 43.1 (C_{20}), 42.7 (C_{14}), 41.0 (C_8), 39.5 (C_1), 39.0 (C_{22}), 37.9 (C_{10}), 33.7 (C_7), 29.6 (C_{15}), 27.4 (C_2), 27.3 (C_{21}), 27.1 (C_{29}), 26.6 (C_{16}), 24.9 (C_{27}), 24.7 (C_{11}), 19.2 (C_6), 17.5 (C_{26}), 16.6 (C_{30}), 16.3 (C_{25}), 12.7 (C_{24}).

Pedunculoside (3): White needles, m.p. 212-213 °C; ESI-MS m/z: 649.3 $[M-H]^-$; ^{1}H-NMR (500 MHz, DMSO-d_6) δ_H (ppm): 5.17 (1H, *d*, *J* = 8.0 Hz, Glc-$H_{1'}$), 4.14 (1H, *d*, *J* = 5.0 Hz, C_{23}-H_α), 3.71 (1H, *s*, C_{23}-H_β), 3.61 (1H, *dd*, *J* = 11.0, 5.5 Hz, C_3-H), 3.60 (1H, *dd*, J= 11.0; 5.5 Hz, $C_{6'}$-H_β), 3,45 (1H, *m*, $C_{6'}$-H_α), 3.19 (1H, *m*, $C_{3'}$-H), 3.13 (1H, *m*, $C_{5'}$-H), 3.10 (1H, *m*, $C_{4'}$-H), 3.08 (1H, *m*, $C_{2'}$-H),1.28 (3H, *s*, C_{27}-H), 1.19 (3H, *s*, C_{29}-H), 0.87 (3H, *s*, C_{25}-H), 0.67 (3H, *s*, C_{26}-H), 0.54 (3H, *s*, C_{24}-H); ^{13}C-NMR (125 MHz, DMSO-d_6) δ_C (ppm): 175.6 (C_{28}), 138.2 (C_{13}), 127.1 (C_{12}), 94.1 ($C_{1'}$), 77.6 ($C_{5'}$), 76.7 ($C_{3'}$), 72.3 ($C_{2'}$), 71.7 (C_{19}), 70.5 (C_3), 69.6 ($C_{4'}$), 64.6 (C_{23}), 60.7 ($C_{6'}$), 53.2 (C_{18}), 47.4 (C_{17}), 46.8 (C_5), 46.5 (C_9), 41.8 (C_4), 41.4 (C_{14}), 41.2 (C_{20}), 39.5 (C_8), 38.1 (C_1), 36.7 (C_{22}), 36.2 (C_{10}), 32.2 (C_7), 28.0 (C_{15}), 26.6 (C_2), 26.4 (C_{29}), 25.8 (C_{16}), 25.1 (C_{21}), 23.2 (C_{11}), 23.9 (C_{27}), 17.8 (C_6), 16.6 (C_{26}), 16.0 (C_{27}), 15.0 (C_{30}), 12.6 (C_{24}).

Uvaol (4): White amorphous powder, m.p. 230-231 °C; ESI-MS: m/z 443 $[M+H]^+$; ^{1}H-NMR (500 MHz, $CDCl_3$) δ_H (ppm): 5.14 (1H, *t*, *J* = 3.0 Hz, C_{12}-H), 3.54 (1H, *d*, *J* = 11.0 Hz, C_{28}-H_α), 3.23 (1H, *dd*, *J* = 11.0, 5.0 Hz, C_3-H), 3.19 (1H, *d*, *J* = 11.0 Hz, C_{28}-H_β), 1.10 (3H, *s*, C_{27}-H), 0.99 (3H, *s*, C_{24}-H), 0.98 (3H, *s*, C_{26}-H), 0.94 (3H, *s*, C_{25}-H), 0.93 (3H, *d*, *J* = 5.2 Hz, C_{30}-H), 0.80 (3H, *d*, *J* = 5.5 Hz, C_{29}-H), 0.79 (3H, *s*, C_{23}-H); ^{13}C-NMR (125 MHz, $CDCl_3$) δ_C (ppm): 138.7 (C_{13}), 125.1 (C_{12}), 79.0 (C_3), 69.7 (C_{28}), 55.3 (C_5), 54.0 (C_{18}), 47.7 (C_9), 42.1 (C_{14}), 40.0 (C_8), 39.5 (C_{20}), 39.4 (C_{19}), 38.8 (C_1), 38.0 (C_4), 36.9 (C_{10}), 36.9 (C_{17}), 32.8 (C_7), 30.8 (C_{21}), 30.6 (C_{15}), 30.5 (C_{22}), 28.1 (C_{23}), 27.3 (C_2), 23.4 (C_{11}), 23.3 (C_{27}), 22.6 (C_{16}), 21.3 (C_{30}), 18.3 (C_6), 17.3 (C_{29}), 16.8 (C_{26}), 15.7 (C_{25}), 15.6 (C_{24}).

Ursolic acid (5): White powder; m.p. 285-286 °C; ESI-MS *m/z*: 457 $[M+H]^+$; ^{1}H-NMR (500 MHz, CD_3OD) δ_H (ppm): 3.21 (1H, *dd, J* = 5.5; 10.5 Hz, C_3-H), 5.24 (1H, *br s*, C_{12}-H), 2.20 (1H, *d, J*=11 Hz, C_{18}-H), 1.09 (3H, *s*, C_{23}-H), 0.78 (3H, *s*, C_{24}-H), 0.98 (3H, *s*, C_{25}-H), 0.85 (3H, *s*, C_{26}-H), 0.93 (2x3H, *s*, C_{27}-H, C_{30}-H), 0.81 (3H, *s*, C_{29}-H); ^{13}C-NMR (125 MHz, CD_3OD) δ_C (ppm): 180.7(C_{28}), 138.1(C_{13}), 125.5(C_{12}), 78.9(C_3), 55.2(C_5), 52.7 (C_{18}), 47.7 (C_{17}), 47.5(C_9), 42.0(C_{14}), 39.4(C_8), 39.0(C_{19}), 38.8(C_{20}), 38.7(C_4), 38.6(C_1), 36.7(C_{22}), 36.9(C_{10}), 33.0(C_7), 30.6(C_{21}), 28.0(C_{23}), 27.9(C_{15}), 26.9(C_2), 24.1(C_{16}), 23.5(C_{27}), 23.2(C_{11}), 21.1(C_{30}), 18.3(C_6), 16.9(C_{26}), 16.8(C_{29}), 15.5(C_{24}), 15.4(C_{25}).

Gypenoside XVII (6): White powder; ESI-MS *m/z*: 945 $[M-H]^-$; ^{1}H-NMR (500 MHz, CD_3OD) δ_H (ppm): 5.15 (1H, *t*, *J* = 7.0 Hz, C_{24}-H), 4.59 (1H, *d*, *J* = 8.0 Hz, $C_{1''}$-H), 4.33 (1H, *t*, *J* = 7.0 Hz, C $_{1'}$-H), 4.32 (1H, *t*, J = 7.0 Hz, $C_{1'''}$-H), 4.03 (1H, *dd*, J = 11.5; 2.0 Hz, $C_{6''}$-H), 3.87 (1H, *dd*, *J* = 11.5; 5.0 Hz, $C_{6'}$-H), 3.85 (1H, *dd*, *J* = 11.5; 5.0 Hz, $C_{6'''}$-H), 3.76 (1H, *dd*, *J* = 11.5; 6.5 Hz, $C_{6''}$-H), 3.75 (1H, *m*, $C_{4'''}$-H), 3.68 (1H, *dd*, *J* = 12.0; 5.0 Hz, $C_{6'''}$-H), 3.49 (1H, *m*, C_{12}-H), 3.42 (1H, *m*, $C_{4'}$-H), 3.37 – 3.31 (6H, *m*, $C_{3'/5'/3''/4''/5''/3'''}$-H), 3.27 – 3.13 (6H, *m*, $C_{3/2'/6'/2''/2'''/5'''}$-H), 2.30 (1H, *m*, C_{17}-H), 2.18 (1H, *m*, C_{23}-H), 2.09 (1H, *m*, C_{23}-H), 1.96 (1H, *m*, C_2-H), 1.91 (1H, *m*, C_2-H), 1.82 – 1.77 (3H, *m*, $C_{13/15/22}$-H), 1.75 – 1.72 (2H, *m*, $C_{1/16}$-H), 1.71 (3H, *s*, C_{26}-H), 1.65 (3H, *s*, C_{27}-H), 1.62 – 1.57 (3H, *m*, $C_{6/7/11}$-H), 1.55 – 1.45 (3H, *m*, $C_{6/9/22}$-H), 1.39 (3H, *s*, C_{21}-H), 1.36 – 1.30 (3H, *m*, $C_{7/15/16}$-H), 1.06 – 1.02 (2H, *m*, $C_{1/11}$-H), 1.07 (3H, *s*, C_{28}-H), 1.03 (3H, *s*, C_{18}-H), 0.95 (3H, *s*, C_{30}-H), 0.94 (3H, *s*, C_{19}-H), 0.88 (3H, *s*, C_{29}-H), 0.81 (1H, *d*, *J* = 11.0 Hz, C_5-H); ^{13}C-NMR (125 MHz, CD_3OD) δ_C (ppm): 132.2 (C_{25}), 126.1 (C_{24}), 106.7 ($C_{1'}$), 105.5 ($C_{1'''}$), 98.1 ($C_{1''}$), 90.6 (C_3), 85.0 (C_{20}), 78.6 ($C_{3''}$), 78.4 ($C_{5'/3'''}$), 78.3 ($C_{3'}$), 77.7 ($C_{5'''}$), 77.5 ($C_{5''}$), 76.6 ($C_{4'}$), 75.6 ($C_{2'''}$), 75.3 ($C_{2''}$), 74.8 ($C_{2'}$), 71.7 ($C_{4'''}$), 71.5 ($C_{4''}$), 71.2 (C_{12}), 70.1 ($C_{6''}$), 66.8 ($C_{6'}$), 62.8 ($C_{6'''}$), 57.6 (C_5), 52.9 (C_{17}), 52.4 (C_{14}), 51.1 (C_9), 49.7 (C_{13}), 41.0 (C_8), 40.3 (C_4), 40.2 (C_1), 37.9 (C_{10}), 36.8 (C_{22}), 35.9 (C_7), 31.5 (C_{11}), 30.9 (C_{15}), 28.4 (C_{28}), 27.3 (C_{16}), 27.2 (C_2), 25.9 (C_{26}), 23.8 (C_{23}), 22.4 (C_{21}), 19.3 (C_6), 18.0 (C_{27}), 17.4 (C_{30}), 16.8 (C_{29}), 16.7 (C_{19}), 16.3 (C_{18}).

Pterocarpin (**7**): Colorless needles m.p. 155-156 °C; ESI-MS *m/z*: 299.3 $[M+H]^+$; ^{1}H-NMR (500 MHz, $CDCl_3$) δ_H (ppm): 7.40 (1H, *d*, *J* = 8,5 Hz, C_1-H), 6.72 (1H, *s*, C_7-H), 6.63 (1H, *dd*, *J* = 2.5; 11.5 Hz, C_2-H), 6.47 (1H, *d*, *J* = 2.5 Hz, C_4-H), 6.43 (1H, *s*, C_{10}-H), 5.90 (2H, *s*, OCH_2O), 5.48 (1H, d, *J* = 7.0 Hz, C_{11a}-H), 4.22 (1H, *dd*, *J* = 5.0; 11.0 Hz, C_6-H), 3.79 (3H, *s*, OCH_3), 3.66 (1H, *t*, J = 8.0 Hz, C_6-H), 3.48 (1H, *dd*, *J* = 5.0; 11.0 Hz, C_{6a}-H) ; ^{13}C-NMR (125 MHz, $CDCl_3$) δ_C (ppm): 161.1 (C_3), 156.6 (C_{4a}), 154.3 (C_{10a}), 148.1 (C_9), 141.7 (C_8), 131.8 (C_1), 117.9 (C_{6b}), 112.4 (C_{11b}), 109.2 (C_2), 104.7 (C_7), 101.7 (C_4), 101.3 (OCH_2O), 93.8 (C_{10}), 78.5 (C_{11a}), 66.5 (C_6), 55.4 (OCH_3), 40.3 (C_{6a}).

Syringin (8): White amorphous powder, m.p. 192-193 °C; ESI-MS *m/z*: 373 $[M+H]^+$; ^{1}H-NMR (500 MHz, CD_3OD) δ_H (ppm): 6.72 (2H, *s*, $C_{3/5}$-H), 6.48 (1H, *d*, *J* = 16.0 Hz, C_7-H), 6.35 (1H, *dt*, *J* = 11.5; 5.0 Hz, C_8-H), 4.91 (1H, *d*, *J* = 7.5 Hz, $C_{1'}$-H), 4.30 (2H, *dd*, *J* =11.5; 5.5 Hz, C_9-H), 3.76 (6H, *s*, $2xCH_3O$), 3.01-3.73 (6H, *m*, $C_{2'/3'/4'/5'/6'}$-H); ^{13}C-NMR (125 MHz, CD_3OD) δ_C (ppm): 152.7 ($C_{2/6}$), 133.9 (C_1), 132.7 (C_4), 130.2 ($C_{1'}$), 128.5 ($C_{2'}$), 104.5 ($C_{1''}$), 102.6 ($C_{3/5}$), 77.2 ($C_{3''}$), 76.6 ($C_{5''}$), 74.2 ($C_{2''}$), 70.0 ($C_{4''}$), 61.5 ($C_{3'}$), 60.9 ($C_{6''}$), 56.4 ($C_{2/6}$-OCH_3)

Daidzin (9): Brown yellow powder, m.p. 245-246 °C; ESI-MS: m/z 417.2 $[M+H]^+$; ^{1}H-NMR (500 MHz, $CDCl_3$) δ_H (ppm): 8.05 (1H, *d*, *J* = 9.0 Hz, C_8-H), 7.23 (1H, *J* = 2.0 Hz, C_6-H), 7,15 (1H, *J* = 1.5 Hz, C_5-H), 7.41 (2H, *d*, *J* = 8.0 Hz, $C_{2'/6'}$-H), 6.82 (2H, *d*, *J* = 8.0 Hz, $C_{3'/5'}$-H); ^{13}C-NMR (125 MHz, $CDCl_3$) δ_C (ppm): 174.7 (C_4), 161.3 (C_7), 157.2 ($C_{4'}$),

157.0 (C_9), 152.2 (C_2), 130.0 ($C_{2'/6'}$), 126.9 (C_5), 123.7 ($C_{1'}$), 122.3 (C_3), 118.5 (C_{10}), 115.6 (C_6), 115.0 ($C_{3'/5'}$), 103.4 (C_8), 100.0 ($C_{1''}$), 77.2 ($C_{5''}$), 76.5 ($C_{3''}$), 73.1 ($C_{2''}$), 69.6 ($C_{4''}$), 60.6 ($C_{6''}$).

Rutin (10): Yellow powder, m.p. 189-190 °C; ESI-MS *m/z* 611 $[M+H]^+$; ^{1}H-NMR (500 MHz, DMSO-d_6) δ_H (ppm): 7.59 (1H, *dd*, *J* = 9.0; 2.1 Hz, $C_{6'}$-H), 7.54 (1H, *d*, *J* = 2.1 Hz, $C_{2'}$-H), 6.85 (1H, *d*, *J* = 9.0 Hz, $C_{5'}$-H), 6.40 (1H, *d*, *J* = 2.0 Hz, C_8-H), 6.20 (1H, *d*, *J* = 2.0 Hz, C_6-H), 5.32 (1H, *d*, *J* = 7.2 Hz, $C_{1''}$-H), 4.39 (1H, *d*, *J* = 1.6 Hz, $C_{1'''}$-H), 3.83–3.35 (10H, *m*, H-$_{2''}$-6ab'' và $_{2'''-5'''}$), 0.99 (3H, *d*, *J* = 6.2); ^{13}C-NMR (125 MHz, DMSO-d_6) δ_C (ppm): 178.2 (C-$_4$), 164.3 (C-$_7$), 161.5 (C-$_5$), 157.1 (C-$_9$), 156.7(C-$_2$), 148.7 (C-$_{4'}$), 145.6 (C-$_{3'}$), 134.1 (C-$_3$), 121.9 (C-$_{1'}$), 122.0 (C-$_{6'}$), 116.5(C-$_{5'}$), 116.1(C-$_{2'}$), 104.2 (C-$_{10}$), 101.4 (C-$_{1''}$), 101.0 (C-$_{1'''}$), 98.9 (C-$_6$), 93.9 (C-$_8$), 76.7 (C-$_{3''}$), 76.7 (C-$_{5''}$), 74.9 (C-$_{2''}$), 72.7 (C-$_{4''}$), 71.4 (C-$_{4'''}$), 71.2 (C-$_{3'''}$), 70.8 (C-$_{2'''}$), 68.5 (C-$_{5'''}$), 67.3 (C-$_{6''}$), 18.0 (C-$_{6'''}$).

Compound **1** was obtained as white powder, mp. 261–262 °C. The ESI-MS of **1** indicated its molecular formula as $C_{30}H_{50}O$ (m/z 427 $[M+H]^+$). Analysis of the ^{1}H-, ^{13}C-NMR, and HSQC spectrum of **1** suggested its friedelane skeleton, which showed the represence of 8 methyl main groups at δ_H 1.18 (3H, s, C_{28}-H)/ δ_C 32.1 (C_{28}), 1.05 (3H, s, C_{27}-H)/ 18.7 (C_{27}), 1.01 (3H, s, C_{26}-H)/ 20.3 (C_{26}), 1.00 (3H, s, C_{29}-H)/ 35.0 (C_{29}), 0.95 (3H, s, C_{30}-H)/ 31.8 (C_{30}), 0.88 (3H, d, *J* = 6.5 Hz, C_{23}-H)/ 6.8 (C_{23}), 0.87 (3H, s, C_{25}-H)/ 18.0 (C_{25}), 0.73 (3H, s, C_{24}-H)/ 14.7 (C_{24}), along with a typical proton signal at δ_H 2.25 (1H, q, *J* = 7.0 Hz, C_4-H)/ δ_C 58.3 (C_4). The ^{13}C-NMR and DEPT of 1 collected 30 signals, comprising 8 primary carbons (CH_3), 11 secondary carbons (CH_2), 4 tertiary carbons (CH), and 7 quaternary carbons (including a carbonyl group C-3 at δ_C 213.6), which revealed the characteristic of a friedelan-3-one triterpene structure. In addition, long-range HMBC correlations of δ_H 0.88 (3H, d, *J* = 6.5 Hz, C_{23}-H) with δ_C 58.3 (C_4), 213.2 (C_3), as well as δ_H 0.73 (3H, s, C_{24}-H) with δ_C 59.5 (C_{10}), 42.2 (C_5), 58.3 (C_4), and 41.3 (C_6), confirmed the basic skeleton. Furthermore, the other characteristic correlation peaks of friedelin were observed in the HMBC spectrum of **1**, including δ_H 1.01 (3H, s, C_{26}-H) to δ_C 39.7 (C_{13}), 38.3 (C_{14}); 1.18 (3H, s, C_{28}-H) to 42.8 (C_{18}), 39.3 (C_{22}), 36.0 (C_{16}), 30.0 (C_{17}); 1.00 (3H, s, C_{29}-H) and 0.95 (3H, s, C_{30}-H) to 35.4 (C_{19}), 32.8 (C_{21}), respectively. The comparison of these signals of **1** with those friedelin reported in the literature is very similar [1]. Friedelin was announced to be isolated from the leaves of species *Phyllanthus reticulatus*, *Hypericum ascyron*, and *Tovomita brasiliensis*, and it has also been demonstrated to be hepatoprotective, antifeedant, and anti-inflammatory without cytotoxicity [2].

Compound **2** was obtained as a white amorphous powder. The ESI-MS of compound **2** indicated its molecular formula as $C_{30}H_{48}O_5$ ($[M+H]^+$ *m/z* 427). The values analysis of ^{1}H-NMR spectrum displayed signals assignable to an olefinic proton δ_H 5.16 (1H, *d*, *J* = 3.5 Hz, C_{12}-H), eighteen protons of six tertiary methyl groups [δ_H 0.70 (*s*), 0.85 (*s*), 1.15 (*d*, *J*=6.5 Hz), 1.29 (*s*), 1.52 (*s*), and 1.60 (*s*) (each, 3H)], two oxymethylene protons [δ_H 3.71 (*s*), 4.13 (*s*)], an oxymethine proton at δ_H 4.38 (1H, *m*, C_3-H). The position of protons exhibited at HSQC correlations at δ_C 12.7 (C_{24}), 17.5 (C_{26}), 16.3 (C_{25}), 27.1 (C_{29}), 24.9 (C_{27}), 16.6 (C_{30}), 67.6 (C_{23}), 74.2(C_3), respectively. The ^{13}C-NMR, DEPT spectrum of compound **2** indicated 30 resonance signals, including a carbonyl carbon (δ_C 182.3); an olefinic quaternary carbon (δ_C 140.0) and an olefinic methine carbon (δ_C 129.5); an oxygenated methine carbon (δ_C 74.2), an oxygenated

quaternary carbon (δ_C 73.6), and an oxygenated methylene carbon (δ_C 67.6); six methyl groups. The complete assignment of all resonances of **2** was revealed by the detailed analysis of HSQC and HMBC. The $^{23}J_{CH}$ correlations at methyl protons (δ_H 0.70) and oxymethylene protons (4.13 (1H, *s*, C_{23}-H_α) and 3.71 (1H, *s*, C_{23}-H_β)) to C_3 (δ_C 74.2)/C_4 (δ_C 43.3) indicated that the position of oxygenated methine carbon (C_3) in the A ring, and oxygenated methylene carbon (C_{23}), methyl carbon (C_{24}) linked C_4 of this ring. In addition, the position of oxygenated quaternary carbon (C_{19}) located the E ring, carbonyl carbon (δ_C 182.3, C_{28}) sited at C_{17}, and tri-substituted double bond at C_{12}/C_{13}, which exhibited the long-range correlations from δ_H 3.09 (1H, *t*, *J* = 7.5; 10.5 Hz, C_{18}-H) to C_{12} (δ_C 129.5)/C_{13} (δ_C 140.0)/C_{28} (δ_C 182.3)/C_{19} (δ_C 73.6). Consequently, compound **2** was established as 3,19,23-trihydroxy-urs-12-en-28-oic acid, namely rotundic acid [2,3].

Compound **3** was isolated as white needles and its mass spectral data suggested the molecular formula as $C_{36}H_{58}O_{10}$. A careful detailed analysis of the data spectrum recorded from compound **3** with the **2** has shown their carbon skeleton similarity as urs-12-en-28-oic acid. The main differences of structure between 3 and **2** were the appearance a *β*-glucosyl unit [δ_H 5.17 (1H, d, *J* = 8.0 Hz, $C_{1'}$-H)/δ_C 94.1 ($C_{1'}$), 3.08 (1H, *m*, $C_{2'}$-H)/72.3 ($C_{2'}$), 3.19 (1H, *m*, $C_{3'}$-H)/76.7 ($C_{3'}$), 3.10 (1H, *m*, $C_{4'}$-H)/69.6 ($C_{4'}$), 3.13 (1H, *m*, $C_{5'}$-H)/77.6 ($C_{5'}$), 3.60 (1H, *dd*, *J*= 11.0; 5.5 Hz, $C_{6'}$-H_β), 3.45 (1H, *m*, $C_{6'}$-H_α)/60.7 ($C_{6'}$)], while the chemical shift of C_{28} changed from δ_C 182.3 to 175.6, which suggested a linked ester between carbonyl carbon and *β*-glucosyl unit in the structure of compound **3**. The carbon signals of the sugar moiety were consistent with those of glucose. In addition, the location of the *β*-glucosyl unit was displayed by the HMBC correlations from $C_{1'}$-H to C_{28}. Base on this evidence and comparison of the data spectrum recorded from compound **3** with the before reported [4], the structure of compound **3** could be assigned as pedunculoside.

Compound **4** was also obtained by using column chromatography of chloroform extract as white amorphous powder, m.p 230-231 °C. ESI-MS $[M+H]^+$ at m/z 443, suggesting the possibility of triterpenoid of molecular formula ($C_{30}H_{50}O_2$). The resonance signals of 1D- and 2D-NMR of compound **4** showed its carbon skeleton similarity as compounds **2** and **3**, which indicated a structural feature of urs-12-ene. The ^{1}H-, ^{13}C-NMR and HSQC spectra also did not record the presence of oxygenated quaternary carbon, carbonyl carbon. Instead, the appearance of resonance signals at δ_H 0.79 (3H, s)/δ_C 28.1, δ_H 0.79 (1H, m)/δ_C 39.4 suggested an additional methyl group and methine carbon. The HMBC spectrum of compound **4** has shown the correlations of the signal at δ_H 0.79 (3H, s) to δ_C 79.0 (C_3)/38.0 (C_4); at H-19 to 54.0 (C_{18})/36.9 (C_{17}). Therefore, the location of methyl group was elucidated link to at C-4, while methine carbon sited C_{19} in *E* ring. In addition, the other difference of compound **4** was the position of oxygenated methylene carbon, which attached to C_{17}. It was elucidated the correlations of the signal at δ_H 3.54 (1H, *d*, *J* = 11.0 Hz, C28-H_α), 3.19 (1H, *d*, *J* = 11.0 Hz, C_{28}-H_β) to 36.9 (C_{17}). Based on the aforementioned data and by comparison with the reported literature was elucidated as urs-12-ene-3β,28-diol (uvaol). Uvaol was previously isolated from *Apocynum venetum* and *Olea europaea* [5,6].

Compound **5**, a white crystalline solid. The molecular formula was determined to be $C_{30}H_{48}O_3$ based on the ESI-MS revealing a molecular ion peak at *m/z* 457

[M+H]$^+$ (calculated for $C_{30}H_{47}O_3$, 457). All over the resonance signals for **5** were in perfect indicated its structure as ursolic acid. Structural characterizations were recorded by using a combination of 1D-NMR (^{1}H, ^{13}C, and DEPT) and 2D-NMR (HSQC, HMBC, COSY, and NOSEY) experiments. Their data revealed the appearance of 7 methyl groups (δ_c 15.4, 16.8, 16.9, 17.0, 21.1, 23.3, and 28.3 ppm), 9 methylene carbons (δ_c 18.0, 22.9, 23.9, 27.0, 27.6, 30.2, 32.7, 36.4, and 38.8 ppm), 5 methine carbons (δ_c 38.5, 38.6, 47.1, 52.4, and 54.8 ppm). In addition, the resonance signals of two carbons at δ_c 76.9 and 124.6 ppm assigned oxygenated methine carbon (C_3) and olefinic (C_{12}) carbon, respectively. Furthermore, the most downfield signal at δ_c 178.3 indicated the carboxylic function (C_{28}). This carboxylic function linked at C_{17}, which supports that long-range correlations between C_{18}-H to C_{28} by HMBC spectrum. The chemical structure of **5** was identical to that of ursolic acid based on the comparison of its NMR and MS data with the published literature [7].

Compound **6**, a white powder. The molecular formula was determined to be $C_{48}H_{82}O_{18}$ based on the ESI-MS revealing a molecular at *m/z* 945 [M-H]$^-$ (calculated for $C_{48}H_{81}O_{18}$, 945.54). The structure of compound **6** has been confirmed and verified that its 1D- and 2D-NMR. The ^{1}H-NMR spectrum, the protons of 3-*O*-glucopyranosyl, 20-*O*-glucopyranosyl moiety showed at 4.33 (1H, *t*, *J* = 7.0 Hz, $C_{1'}$-H), 4.59 (1H, *d*, *J* = 8.0 Hz, $C_{1''}$-H), 4.32 (1H, *t*, *J* = 7.0 Hz, $C_{1'''}$-H), respectively. The anomeric proton signals has been shown that compound **6** harbored three *β*-D-glucoses moiety. On other hand, a comparison of the ^{13}C-NMR spectrum of compound **6** with that of gypenoside XVII suggested the compound **6** to identical 3-*O*-[*β*-D-glucopyranosyl]-20-*O*-[*β*-D-glucopyranosyl-(6→1)-*β*-D-glucopyranosyl]-20(*S*)-protopanaxadiol [8]. Based on the foregoing observations, compound **6** was elucidated to be gypenoside XVII.

Compound **7** was obtained as colorless needles and its molecular formula was established as $C_{17}H_{14}O_5$ by ESI-MS (*m/z* 299.3 [M+H]$^+$). The ^{1}H-NMR spectrum, compound **7** showed a characteristic set of peaks corresponding to a pterocarpan skeleton; the signals at δ 5.48 (1H, *d*, *J* = 7.0 Hz), 4.22 (1H, *dd*, *J* = 5.0; 11.0 Hz), 3.66 (1H, *t*, *J* = 8.0 Hz), 3.48 (1H, *dd*, *J* = 5.0; 11.0 Hz) were assigned to H-11a, H-6, and H-6a, respectively. The ^{1}H-NMR spectrum also displayed the doublet at δ_H 5.90 (2H, *s*, -OCH_2O-) and two singlets at δ_H 6.43 (1H, *s*, C_{10}-H) and 6.72 (1H, *s*, C_7-H), which suggested compound **7** has a methylenedioxy moiety in the aromatic ring D. Furthermore, it showed a doublet at δ_H 6.47 (1H, *d*, *J* = 2.5 Hz, C_4-H), a doublet of doublets at δ_H 6.63 (1H, *dd*, *J* = 2.5; 11.5 Hz, C_2-H) and a doublet at δ_H 7.40 (1H, *d*, *J* = 8.5 Hz, C_1-H) assignable to the ABX system for the ring A and a methyl group at 3.79 (3H, *s*, OCH_3). The ^{13}C NMR, DEPT and HSQC spectra of compound **7** indicated the signal of 16 carbons, including 1 sp^3 oxygenated tertiary (δ c 90.0), 3 sp^3 methine (δ c 47.7, 66.9, 88.4), 4 sp^3 methylene (δ c 29.7, 36.7, 43.3, 72.9, 101.5), 1 sp^2 quaternary (δ c 117.9), 3 sp^2 oxygenated tertiary (δ c 141.7, 148.1, 156.6), 2 sp^2 methine (δ c 93.2,114.2), 1 methylenedioxy group (δ c 101.3). Moreover, the ^{13}C-NMR chemical shift of **7** was assigned based on further analyses of its HSQC and HMBC spectra. The cross-peaks between H-1/C-3, H-1/C-11a, H-2/C-3, H-2/C-11b, H-4/C-3, H-4/C-11b in the HMBC spectrum confirmed the planar structure of 7. In addition, the signal at δ_H 5.90 (OCH_2O) showed a long-range correlation with C-8 and -9 at δ_C 141.7 and 148.1,

respectively. Based on the foregoing observations, compound **7** was elucidated to be a pterocarpin [9].

Compound **8** was isolated as white amorphous powder and its mass spectral data suggested the molecular formula as $C_{17}H_{24}O_9$. The ^{1}H-NMR spectrum of **1** indicated the presence of phenylpropanoid skeleton at δ_H 6.48 (1H, *d*, *J* = 16.0 Hz, C_7-H), 6.35 (1H, *dt*, *J* = 11.5; 5.0 Hz, C_8-H), 4.30 (2H, *dd*, *J* =11.5; 5.5 Hz, C_9-H) and a glucose moiety at δ_H 3.01-3.73 (6H, *m*, $C_{2'/3'/4'/5'/6'}$-H), an anomeric proton at δ 4.91 (1H, *d*, *J* = 7.5 Hz, $C_{1'}$-H). The coupling constant of *J* = 16.0 Hz was attributable to one pair of *trans* protons which is the hallmark of cinnamic acid derivatives, *m*-substituted aromatic ring system signals were observed at δ_H 6.72 (2H, *s*, $C_{3/5}$-H), and two methoxy groups were revealed at 3.76 (6H, *s*, 2- and 6-OCH_3). In the ^{13}C NMR data, a glucose moiety between δ_C 60.9 ($C_{6''}$) and 76.6 ($C_{5''}$), an anomeric carbon signal at δ_C 104.5 ($C_{1''}$) were confirmed. The signal at δ_C 56.4 ($C_{2/6}$-OCH_3) indicated two methoxy carbons. Finally, the glucosyl C1′-hydrogen atom (δ_H 4.91) correlated with C_1 (δ_C 133.9) of the phenylpropanoid unit in the HMBC spectrum. On the basis of the 1D-, 2D- NMR experiments, compound **8** was assumed as syringin [4].

Compound **9** was obtained as brown-yellow powder. The ESI-MS of compound **9** showed *m*/*z* 417.2 $[M+H]^+$, was established to be $C_{21}H_{21}O_9$. The signals of aromatic protons are registered at δ_H 7.41 (2H, *d*, *J* = 8.0 Hz, $C_{2'/6'}$-H) and 6.82 (2H, *d*, *J* = 8.0 Hz, $C_{3'/5'}$-H). It also exhibited a signals at δ_H 8.05 (1H, *d*, *J* = 9.0 Hz, C_8-H), and 7.23 (1H, *J* = 2.0 Hz, C_6-H). In addition, the proton signal at C-5 at δ_H 7,15 (1H, *J* = 1.5 Hz, C_5-H) resound. Studies on ^{13}C-NMR spectra of flavonoids, carbon chemical shifts of the aglycone skeleton of compound **9** were consistent with these assignments. The signals at δ_C 115.6 and 103.4 were assigned to the C-6 and C-8, respectively. Also, the C-2 signal was considered to be at δ_C 152.2 of the isoflavone. The oxygenated carbon, C-4', appeared at δ_C 157.2 ppm. C-3', 5' and C-2', 6' were assigned to the signals at δ_C 115.0 and 130.0. The ^{13}C NMR chemical shift data of the glucose moiety of daidzin were clearly indicated by analysis of the ^{13}C NMR spectra, especially by the resonances for C-1", C-2", C-3", C-4", C-5" and C-6" (δ_C 100.0, 73.1, 76.5, 69.6, 77.2 and 60.6, respectively). The location of the *β*-glucosyl unit was determined by $_3J$-HMBC correlations between H-1′ to C-7. Thus, the structure of **9** was established as daidzin (daidzein-7-O-*β*-D-glucopyranoside) [10].

Compound **10** was obtained as a yellow powder. The ESI-MS with an $[M+H]^+$ ion at *m*/*z* 611 together with the ^{13}C NMR and HMBC data indicated a molecular formula of $C_{27}H_{30}O_{16}$. The ^{1}H-NMR spectrum of compound **10** was showed the characteristic signals of the quercetin skeleton: δ_H 6.26 (*d*, *J* = 2.1 Hz, C_6-H), 6.45 (d, J = 2.1 Hz, C_8-H), 7.68 (dd, J = 8.4, 2.2 Hz, C_6-H), 6.92 (*d*, *J* = 8.4 Hz, H-5), and 7.71 (*d*, *J* = 2.2 Hz, H-2). In the HMBC spectrum, the rhamnosyl C1-hydrogen atom (δ_H 4.56) correlated with the glucosyl C6′ atom at δ C 68.5 indicating a rutinosyl moiety. Finally, the glucosyl C1′-hydrogen atom (δ_H 5.15) correlated with C3 (δ_C 135.62) of the flavonoid unit in the HMBC spectrum. The analysis of the one- and two-dimensional ^{1}H- and ^{13}C-NMR spectra of **10** and comparison with the values found in the literature [11] led to the assignment of compound **10** as quercetin-3-*O*-rutinoside (rutin).

Table S1. α-Glucosidase inhibition by the isolated compounds from *Millettia speciosa.*

Conc. (μg/mL)	Inhibition (%)										
	**1**[a]	**2**	**3**	**4**	**5**	**6**	**7**	**8**	**9**	**10**	Acarbose[b]
256	23	33	60.5	92.5	99	34	25	12	22	85	72
64	20	4	32	85	98	21	12	9	10	82.5	23
16	13	1	9.5	83	95	17	3	8	4	78	18
4	1	1	3	76.5	91.5	5	3	1	0	74	5
1	1	1	1	37.5	48.5	1	0	0	0	34.5	0

[a] Compounds **1**: friedelin; **2**: rotundic acid; **3**: pedunculoside; **4**: uvaol; **5**: ursolic acid; **6**: gypenoside XVII; **7**: pterocarpin; **8**: syringin; **9**: daidzin, and **10**: rutin. [b] Acarbose was used as a positive control in the evaluation of α-glucosidase inhibition activity.

Table S2. Half-maximal inhibitory concentration (IC_{50}) for acetylcholinesterase inhibition by the isolated compounds from *Millettia speciosa*.

Compound	Half-maximal inhibitory concentration (IC_{50}), μg/mL
	AChE
**1**	>256
**2**	>256
**3**	>256
**4**	>256
**5**	8.0 ± 0.75
**6**	>256
**7**	>256
**8**	>256
**9**	>256
**10**	256 ± 7.89
PC	0.025 ± 0.007

AChE: Acethylcholinesterase. Compounds **1**: friedelin; **2**: rotundic acid; **3**: pedunculoside; **4**: uvaol; **5**: ursolic acid; **6**: gypenoside XVII; **7**: pterocarpin; **8**: syringin; **9**: daidzin, and **10**: rutin. PC: positive controls with donepezil for AChE inhibition.

Detailed description for docking studies

Protein Preparation

Since the crystallographic structure of *Saccharomyces cerevisiae* α-glucosidase enzyme is not available in Protein data bank, so to understand the ligand-enzyme interactions, the three-dimensional structure of α-glucosidase was built by homology modeling on Swiss-Model webserver (https://swissmodel.expasy.org/). The template structure was searched on NCBI protein BLAST to model the protein of interest. Swiss-Model suggested a crystal structure of isomaltase enzyme from *S. cerevisiae* (PDB ID: 3AJ7) with 72.4% identity and 91% query coverage. The stereochemical aspects of the model were inspected by check the Ramachandran plot, it could be considered as a liable model for further docking studies (Figure S3). It is commonly assumed that, a good quality model would be expected to have over 90% in the most favored regions. Obtained data from Ramachandran plot showed that amino acids residues of the α-glucosidase model located in the most favored regions, thus, it could be considered as a liable model for further docking studies. The crystal structure of human intestinal α-glucosidase in complex with acarbose inhibitor (PDB ID: 3TOP) was retrieved from the Protein Data Bank. All water molecules and small molecules were deleted by Discovery.

Ligand Preparation

The three-dimensional structures of compounds were prepared using ChemDraw version 16.0 (PerkinElmer®). The energy minimization was carried out using MM2 force field and quantum chemical calculations were performed by PM6 semiempirical method implemented in GaussView 6.0 and Gaussian 09.

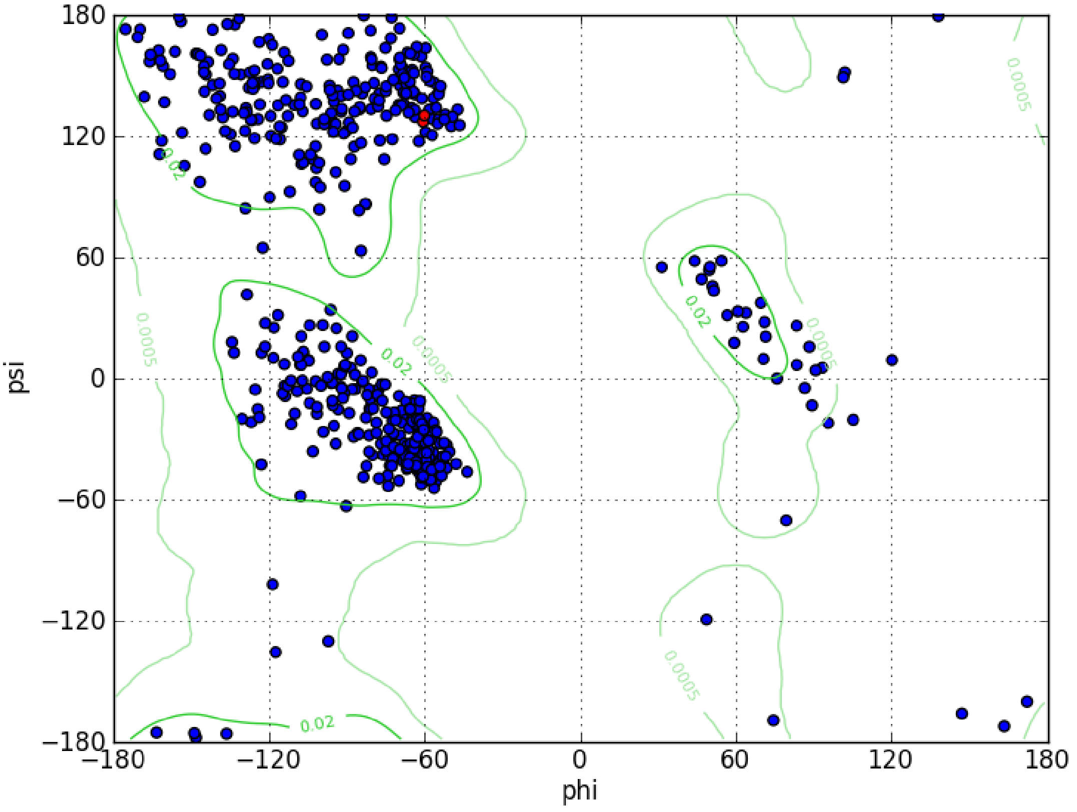

Figure S3. Ramachandran plot analysis of the structure of *Saccharomyces cerevisiae* α-glucosidase model.

Molecular docking

AutoDock Vina was employed to set up and performed docking calculations by the PyRx program. We performed the docking study assuming that having a rigid protein and consider the conformational space of the ligands to analyze the inductive effect of the hybrid compounds. In the docking analysis, the binding site was enclosed in a box with the number of grid points in x × y × z dimensions (25 Å × 25 Å × 25 Å), the center of the grid box was placed at x = 22.2262, y = - 8.1477, z = 23.9431 for *Saccharomyces cerevisiae* α-glucosidase enzyme and x = 22.2262, y = - 8.1477, z = 23.9431 for human intestinal α-glucosidase enzyme. The remaining parameters are kept at the default of the software. Autodock Vina was run to find out 10 sites in which lowest energy estimations between ligand and receptor were given. The

outputs from AutoDock Vina modeling studies were analyzed using Discovery Studio Visualizer.

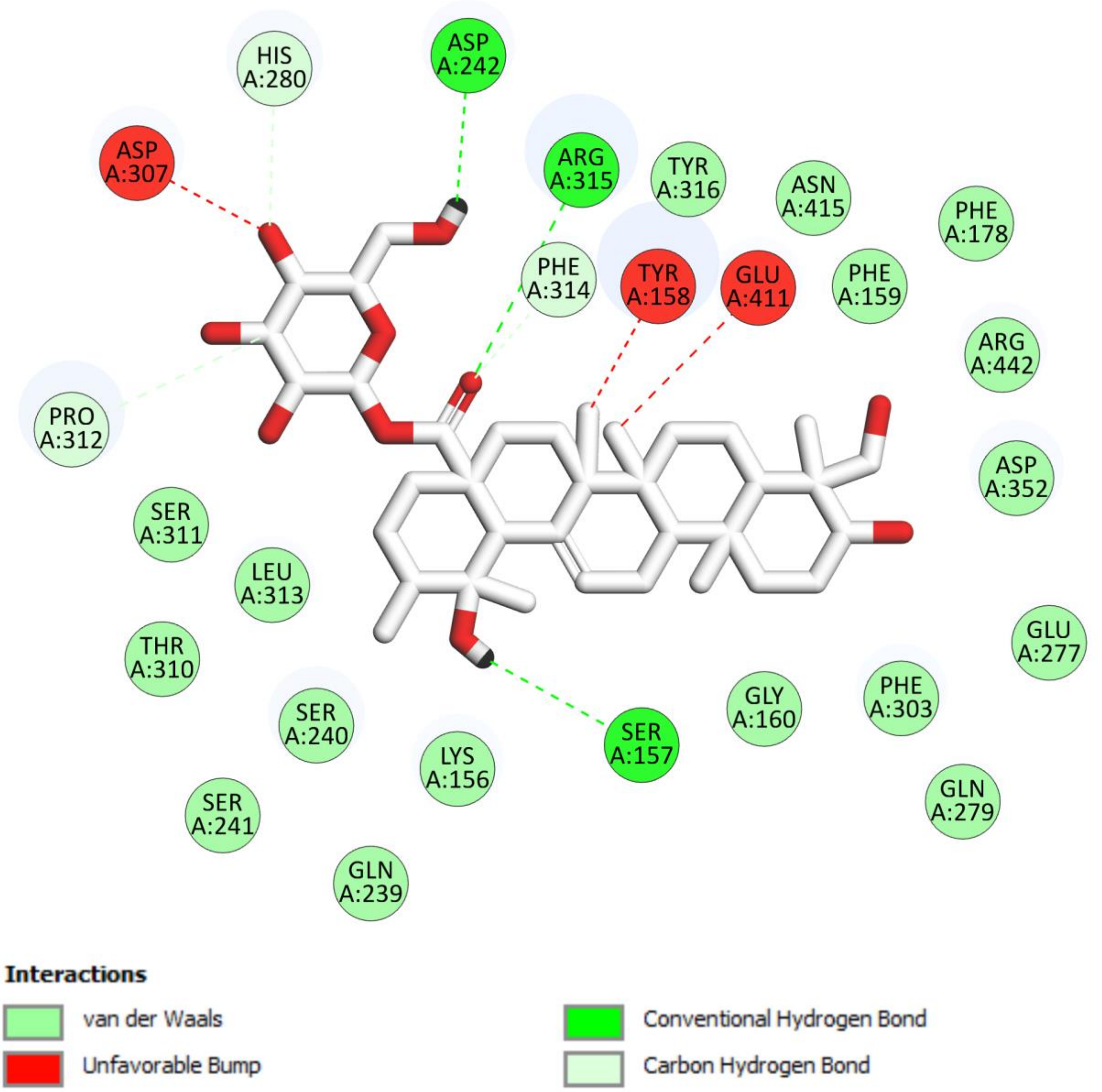

Figure S4. 2D binding model of compound 3 in the active site of *Saccharomyces cerevisiae* α-glucosidase enzyme.

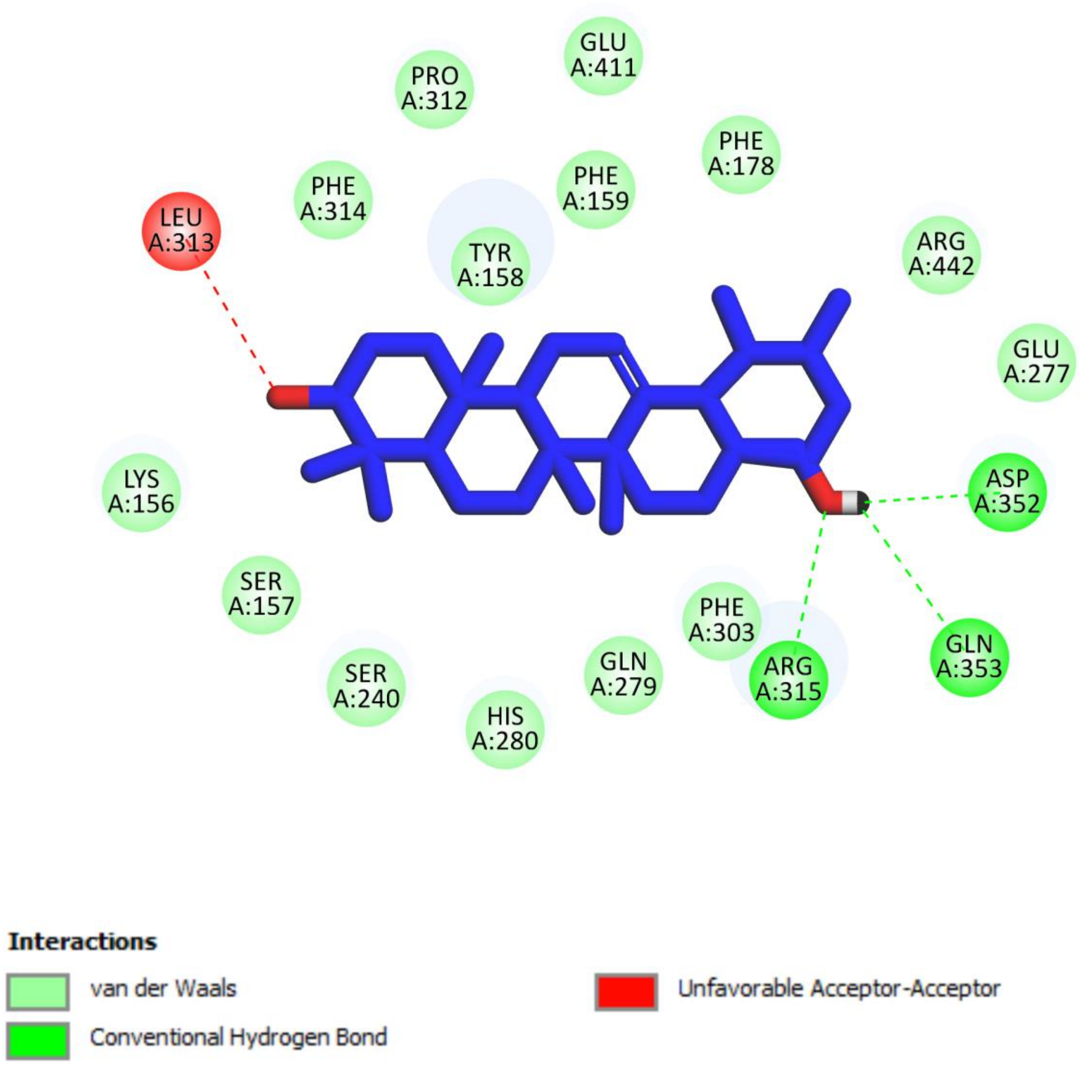

Figure S5. 2D binding model of compound 4 in the active site of *Saccharomyces cerevisiae* α-glucosidase enzyme.

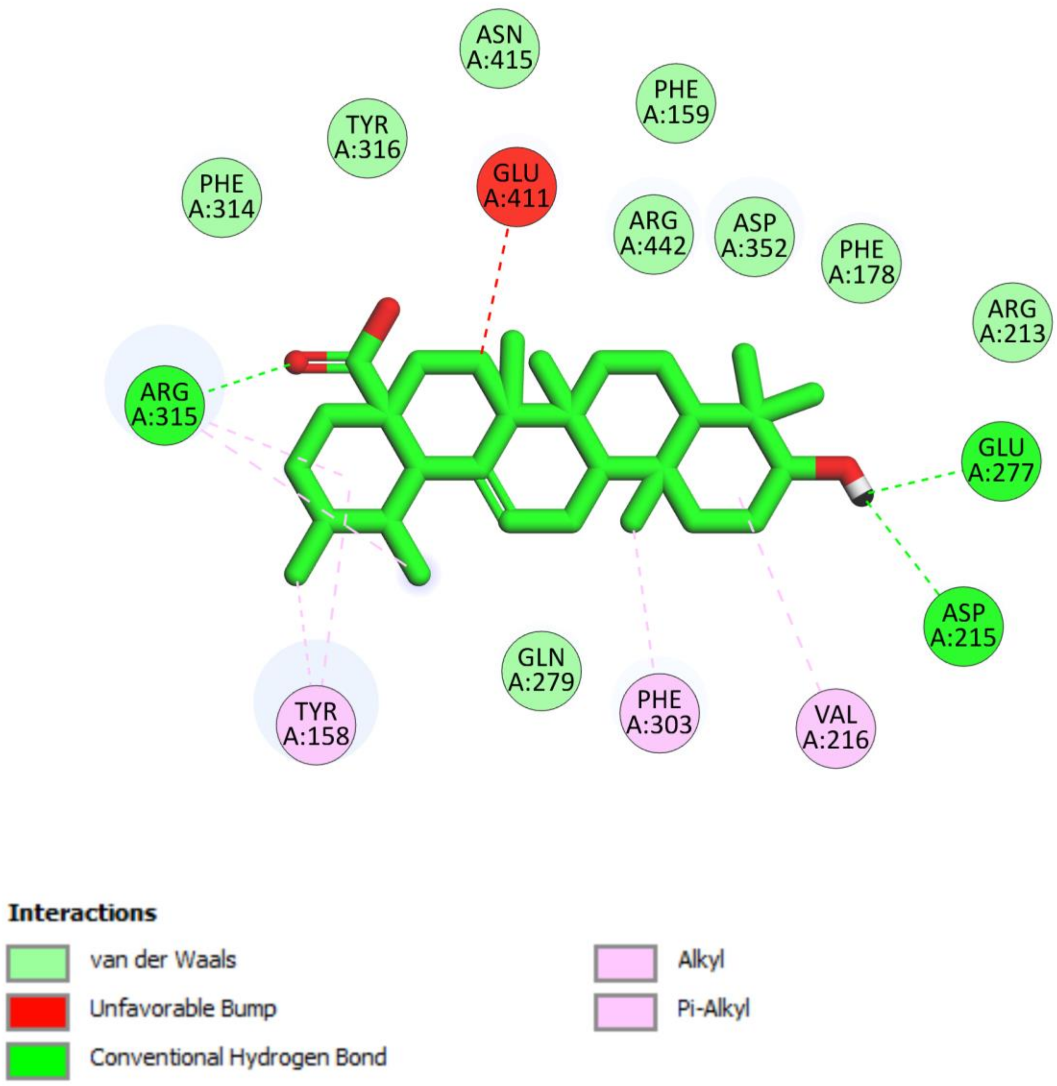

Figure S6. 2D binding model of compound **5** in the active site of *Saccharomyces cerevisiae* α-glucosidase enzyme.

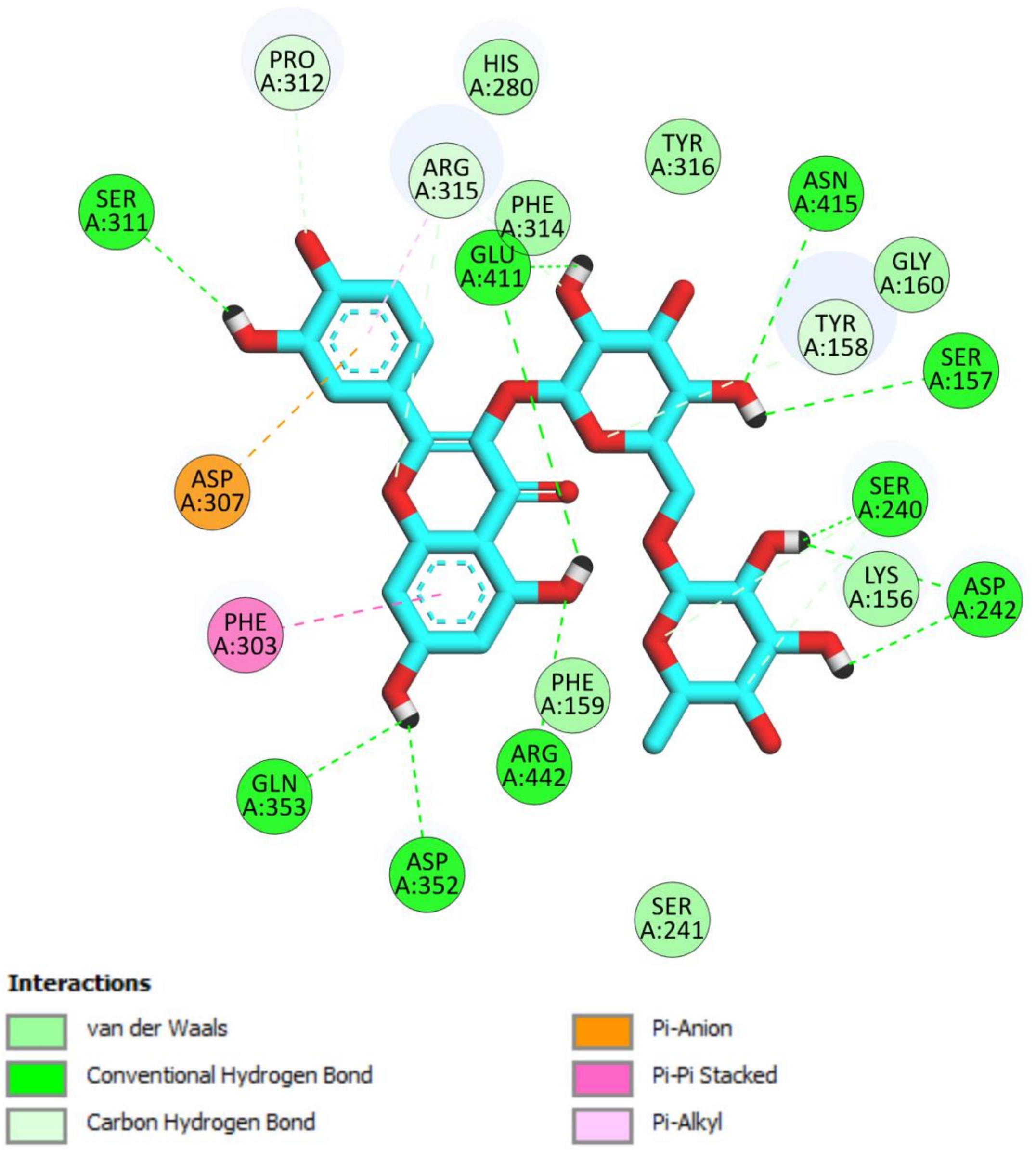

Figure S7. 2D binding model of compound **10** in the active site of *Saccharomyces cerevisiae* α-glucosidase enzyme.

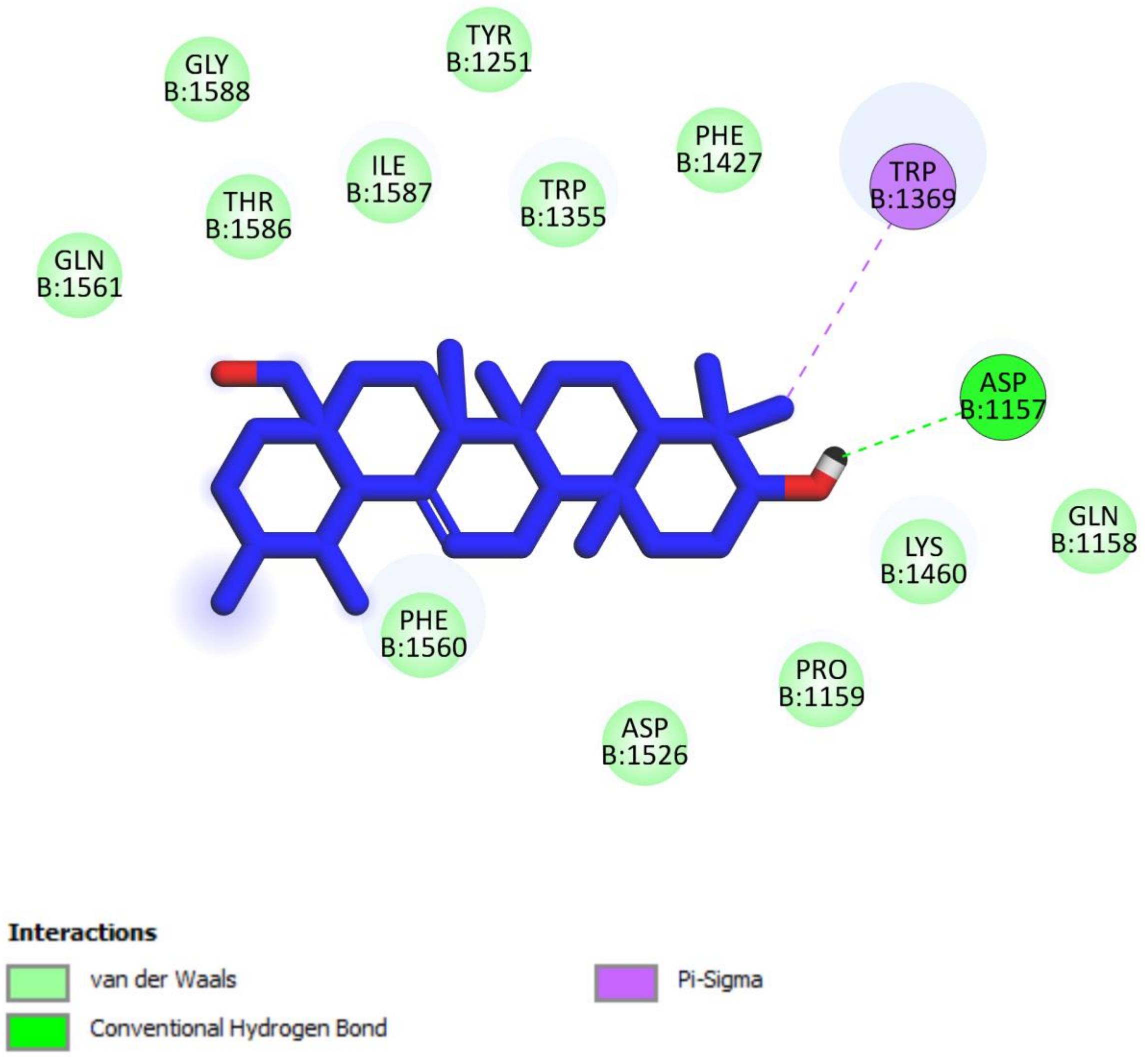

Figure S8. 2D binding model of compound **4** in the active site of human intestinal α-glucosidase enzyme.

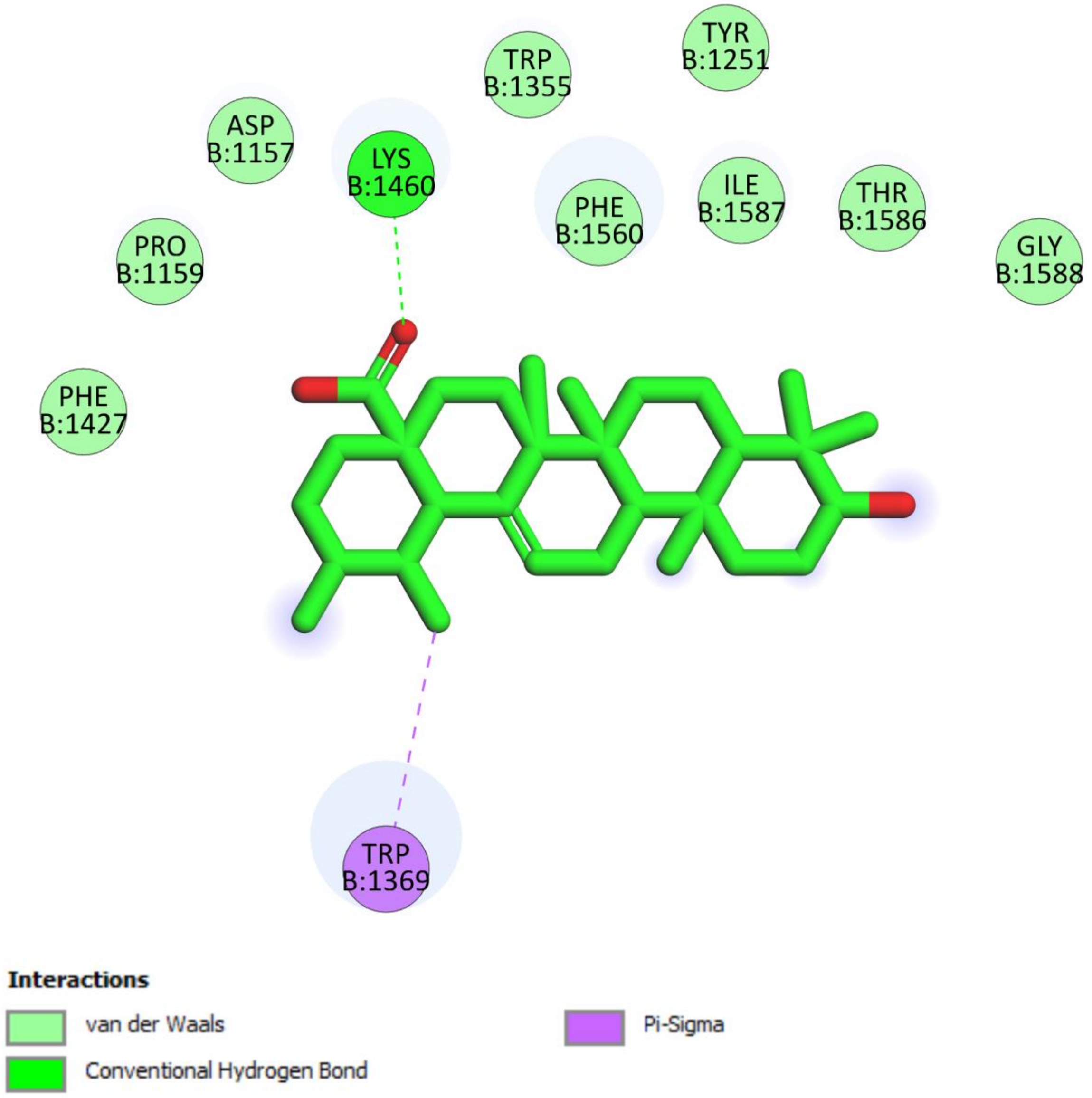

Figure S9 . 2D binding model of compound **5** in the active site of human intestinal α-glucosidase enzyme

References

1. Antonisamy, P.; Duraipandiyan, V.; Ignacimuthu, S. Anti-Inflammatory, Analgesic and Antipyretic Effects of Friedelin Isolated from Azima Tetracantha Lam. in Mouse and Rat Models. *J. Pharm. Pharmacol.* **2011**, *63*, 1070–1077, doi:10.1111/j.2042-7158.2011.01300.x.
2. Nguyen, H.T.; Ho, D.V.; Vo, H.Q.; Le, A.T.; Nguyen, H.M.; Kodama, T.; Ito, T.; Morita, H.; Raal, A. Antibacterial Activities of Chemical Constituents from the Aerial Parts of Hedyotis Pilulifera. *Pharm. Biol.* **2017**, *55*, 787–791, doi:10.1080/13880209.2017.1279673.
3. Aro, A.O.; Dzoyem, J.P.; Awouafack, M.D.; Selepe, M.A.; Eloff, J.N.; McGaw, L.J. Fractions and Isolated Compounds from Oxyanthus Speciosus Subsp. Stenocarpus (Rubiaceae) Have Promising Antimycobacterial and Intracellular Activity. *BMC Complementary Altern. Med.* **2019**, *19*, 108, doi:10.1186/s12906-019-2520-x.
4. Wang, C.; Chao, Z.; Sun, W.; Wu, X.; Ito, Y. Isolation of Five Glycosides from the Barks of Ilex Rotunda by High-Speed Counter-Current Chromatography. *J. Liq. Chromatogr. Relat. Technol.* **2014**, *37*, 2363–2376, doi:10.1080/10826076.2013.832297.
5. Bonel-Pérez, G.C.; Pérez-Jiménez, A.; Gris-Cárdenas, I.; Parra-Pérez, A.M.; Lupiáñez, J.A.; Reyes-Zurita, F.J.; Siles, E.; Csuk, R.; Peragón, J.; Rufino-Palomares, E.E. Antiproliferative and Pro-Apoptotic Effect of Uvaol in Human Hepatocarcinoma HepG2 Cells by Affecting G0/G1 Cell Cycle Arrest, ROS Production and AKT/PI3K Signaling Pathway. *Molecules* **2020**, *25*, E4254, doi:10.3390/molecules25184254.
6. Du, S.-Y.; Huang, H.-F.; Li, X.-Q.; Zhai, L.-X.; Zhu, Q.-C.; Zheng, K.; Song, X.; Xu, C.-S.; Li, C.-Y.; Li, Y.; et al. Anti-Inflammatory Properties of Uvaol on DSS-Induced Colitis and LPS-Stimulated Macrophages. *Chin. Med.* **2020**, *15*, 43, doi:10.1186/s13020-020-00322-0.
7. Ono, M.; Koto, M.; Komatsu, H.; Igoshi, K.; Kobayashi, H.; Ito, Y.; Nohara, T. Cytotoxic Triterpenes and Sterol from the Fruit of Rabbiteye Blueberry (*Vaccinium Ashei*). *Food Sci. Technol. Res.* **2004**, *10*, 56–59, doi:10.3136/fstr.10.56.
8. Cheng, L.-Q.; Na, J.-R.; Kim, M.K.; Bang, M.-H.; Yang, D.-C. Microbial Conversion of Ginsenoside Rb1 to Minor Ginsenoside F2 and Gypenoside XVII by Intrasporangium Sp. GS603 Isolated from Soil. *J. Microbiol. Biotechnol.* **2007**, *17*, 1937–1943.
9. Fukui, K.; Nakayama, M. Syntheses of Pterocarpans. II. The Synthesis of (±)-Pterocarpin. *BCSJ* **1969**, *42*, 1408–1411, doi:10.1246/bcsj.42.1408.
10. Fedoreyev, S.A.; Bulgakov, V.P.; Grishchenko, O.V.; Veselova, M.V.; Krivoschekova, O.E.; Kulesh, N.I.; Denisenko, V.A.; Tchernoded, G.K.; Zhuravlev, Y.N. Isoflavonoid Composition of a Callus Culture of the Relict Tree Maackia Amurensis Rupr. et Maxim. *J. Agric. Food Chem.* **2008**, *56*, 7023–7031, doi:10.1021/jf801227q.
11. Zor, M.; Aydin, S.; Güner, N.D.; Başaran, N.; Başaran, A.A. Antigenotoxic Properties of Paliurus Spina-Christi Mill Fruits and Their Active Compounds. *BMC Complementary Altern. Med.* **2017**, *17*, 229, doi:10.1186/s12906-017-1732-1.
